# Integrative omics reveals mechanisms of biosynthesis and regulation of floral scent in *Cymbidium tracyanum*

**DOI:** 10.1111/pbi.70025

**Published:** 2025-03-17

**Authors:** Mengling Tu, Ningyawen Liu, Zheng‐Shan He, Xiu‐Mei Dong, Tian‐Yang Gao, Andan Zhu, Jun‐Bo Yang, Shi‐Bao Zhang

**Affiliations:** ^1^ Key Laboratory for Economic Plants and Biotechnology, Kunming Institute of Botany Chinese Academy of Sciences Kunming Yunnan China; ^2^ University of Chinese Academy of Sciences Beijing China; ^3^ National Key Laboratory of Genetic Evolution & Animal Models, Kunming Natural History Museum of Zoology, Kunming Institute of Zoology Chinese Academy of Sciences Kunming Yunnan China; ^4^ Germplasm Bank of Wild Species & Yunnan Key Laboratory of Crop Wild Relatives Omics, Kunming Institute of Botany Chinese Academy of Sciences Kunming Yunnan China

**Keywords:** orchid, *cymbidium tracyanum*, multi‐omics, floral scent, terpene synthase

## Abstract

Flower scent is a crucial determiner in pollinator attraction and a significant horticultural trait in ornamental plants. Orchids, which have long been of interest in evolutionary biology and horticulture, exhibit remarkable diversity in floral scent type and intensity. However, the mechanisms underlying floral scent biosynthesis and regulation in orchids remain largely unexplored. In this study, we focus on floral scent in *Cymbidium tracyanum*, a wild species known for its strong floral fragrance and as a primary breeding parent of commercial *Cymbidium* hybrids. We present a chromosome‐level genome assembly of *C. tracyanum*, totaling 3.79 Gb in size. Comparative genomic analyses reveal significant expansion of gene families associated with terpenoid biosynthesis and related metabolic pathways in *C. tracyanum*. Integrative analysis of genomic, volatolomic and transcriptomic data identified terpenoids as the predominant volatile components in the flowers of *C. tracyanum*. We characterized the spatiotemporal patterns of these volatiles and identified *CtTPS* genes responsible for volatile terpenoid biosynthesis, validating their catalytic functions *in vitro*. Dual‐luciferase reporter assays, yeast one‐hybrid assays and EMSA experiments confirmed that *CtTPS2*, *CtTPS3,* and *CtTPS8* could be activated by various transcription factors (i.e., CtAP2/ERF1, CtbZIP1, CtMYB2, CtMYB3 and CtAP2/ERF4), thereby regulating the production of corresponding monoterpenes and sesquiterpenes. Our study elucidates the biosynthetic and regulatory mechanisms of floral scent in *C. tracyanum*, which is of great significance for the breeding of fragrant *Cymbidium* varieties and understanding the ecological adaptability of orchids. This study also highlights the importance of integrating multi‐omics data in deciphering key horticultural traits in orchids.

## Introduction

Orchidaceae is one of the most diverse families of angiosperms, comprising nearly 800 genera and over 29 000 species worldwide (Christenhusz and Byng, 2016; Govaerts *et al*., 2021), many of which are of considerable ornamental and medicinal value. The floral organs of orchids have evolved remarkably diverse morphology, colour and scent, enabling deceptive pollination strategies in nearly one‐third of orchid species (Cozzolino and Widmer, 2005; Jersáková *et al*., 2006). Floral scent is a key horticultural trait for ornamental plants and affects plant adaptation to environmental disturbances, including defence against pathogens and folivores (Holopainen and Gershenzon, 2010; Huang *et al*., 2011), pollinator attraction (Byers *et al*., 2014) and mediating plant‐environment interactions (Heil and Silva Bueno, 2007; Kegge and Pierik, 2010). These complex interactions between environmental disturbances and diversified floral features make orchids invaluable for research in evolutionary biology and horticulture (Pérez‐Escobar *et al*., 2024; Waterman and Bidartondo, 2008).

Floral scent is composed of various volatile organic compounds (VOCs): terpenoids, phenylpropanoids/benzenoids and fatty acid derivatives, based on their biosynthetic origins (Dudareva *et al*., 2013). The most diverse and largest proportion of VOCs is the terpenoids, including terpenes and their modified forms (Chen *et al*., 2011; Tholl, 2015). Terpenoids are synthesized from two common 5‐carbon precursors—isopentenyl diphosphate (IPP) and its allylic isomer, dimethylallyl diphosphate (DMAPP) (McGarvey and Croteau, 1995). These C5‐isoprene units are generated from two independent and compartmentalized pathways. The methylerythritol phosphate (MEP) pathway, which occurs in plastids, produces both IPP and DMAPP. The mevalonic acid (MVA) pathway, which occurs in the cytosol, endoplasmic reticulum and peroxisomes, primarily produces IPP. Prenyltransferases use IPP and DMAPP as substrates to catalyse head‐to‐tail coupling condensation in different proportions to yield prenyl diphosphate precursors—farnesyl diphosphate (FPP) in the cytosol, geranyl diphosphate (GPP) and geranylgeranyl diphosphate (GGPP) in plastids.

Terpenoid precursors are converted into the basic carbon skeletons of monoterpenes, sesquiterpenes and diterpenes by terpene synthases (TPSs) (Chen *et al*., 2011; Jia *et al*., 2022). Because many TPSs are multifunctional and located at key branching points in the isoprenoid pathway, they produce a variety of main products and by‐products, contributing significantly to the structural diversity of terpenoids (Jia *et al*., 2022). Recent biochemical and molecular studies of TPSs have provided significant insights into their evolutionary, structural and mechanistic properties, as well as their regulatory mechanisms. However, functional identification of *TPS* genes in orchids remains limited (Chuang *et al*., 2018; Yu *et al*., 2020).

Synthesis and release of terpenoids in plants is spatially (tissues and organs) and temporally specific (circadian rhythm and developmental stages) (Dudareva *et al*., 2003; Jørgensen *et al*., 2005). Previous studies have shown that higher expression levels of terpenoid biosynthesis‐related genes often correlate with the primary sites of floral scent production (Bergougnoux *et al*., 2007; Nagegowda *et al*., 2008), indicating that the synthesis of floral scent components relies on key transcription factors (TFs) that determine the spatiotemporal expression of enzyme genes (Colquhoun and Clark, 2011). To date, at least six families of TFs have been implicated in the regulation of terpenoid biosynthesis: AP2/ERF, bHLH, bZIP, ARF, MYB and WRKY (Hong *et al*., 2012; Samad *et al*., 2017; Tan *et al*., 2015). For instance, studies have shown that members of the bHLH family regulate sesquiterpene production by directly binding to promoters of the sesquiterpene synthase genes *AtTPS11* and *AtTPS21* (Hong *et al*., 2012). In addition, TPS activity in terpenoid biosynthesis is differentially regulated at the post‐transcriptional or translational levels (Picazo‐Aragonés *et al*., 2020).

Floral VOCs have been characterized (Xu *et al*., 2017) and *TPS* genes have been identified in various orchid species, for example, *Apostasia shenzhenica* (Huang *et al*., 2021; Yang *et al*., 2021; Zhang *et al*., 2016), *Phalaenopsis equestris* (Huang *et al*., 2021; Tsai *et al*., 2017; Yang *et al*., 2021; Zhang *et al*., 2016), *Vanilla planifolia* (Huang *et al*., 2021) and *Dendrobium officinale* (Huang *et al*., 2021; Tsai *et al*., 2017; Yang *et al*., 2021; Zhang *et al*., 2016). In *Cymbidium*, the dominant floral volatiles have been shown to vary between species. For example, the dominant floral volatiles include farnesol, methyl epi‐jasmonate, (*E*)‐β‐farnesene and nerolidol in *C. goeringii* (Ramya *et al*., 2019), whereas they are methyl jasmonate, acacia alcohol and linalool in *C. ensifolium* (Ai *et al*., 2021). The remarkable diversity in both the type and intensity of fragrance across *Cymbidium* species makes this genus ideal for examining the evolution of molecular mechanisms that regulate floral scent.

*Cymbidium* are world‐renowned ornamental orchids, with terrestrial species (commonly known as Chinese orchids) cultivated in China for over 1000 years. These orchids are highly prized for their fragrant flowers, diverse flower shapes and elegant plant forms in Asian countries (Liu *et al*., 2006). Over the past century, hybrids derived from epiphytic *Cymbidium* species have dominated the global flower market, but artificial selection for other traits has often resulted in a loss of fragrance, even from aromatic parents (Dudareva and Pichersky, 2008; Ramya *et al*., 2019). Importantly, *Cymbidium* includes scentless orchids (e.g., *C. lowianum*), which enable comparative analysis between orchid species to identify VOCs, synthetic enzymes and differentially expressed TFs that may play a role in scent biosynthesis. Furthermore, several *Cymbidium* orchid genomes have recently been sequenced, which facilitate the identification of related genomic changes.

In this study, we presented a chromosome‐level genome assembly of *C. tracyanum*, a species characterized by strongly fragrant flowers with more than 10 flowers per scape, making it a key parent in hybrid breeding. Comparative genome analysis with other orchids revealed a significant expansion of gene families associated with terpenoid biosynthesis. Furthermore, through comparative transcriptomic and metabolomic analyses between the fragrant *C. tracyanum* and the scentless *C. lowianum*, we elucidated the biosynthetic pathways and transcriptional regulation of floral scent. We identified, isolated, cloned and functionally analysed *TPS* genes in *C. tracyanum*, uncovering the potential regulatory network of terpenoid biosynthesis. These findings shed light on the breeding of fragrant varieties and reveal the evolution and adaptation of orchids.

## Results

### Chromosome‐level genome assembly and annotation of *C. tracyanum*

**Figure 1:**
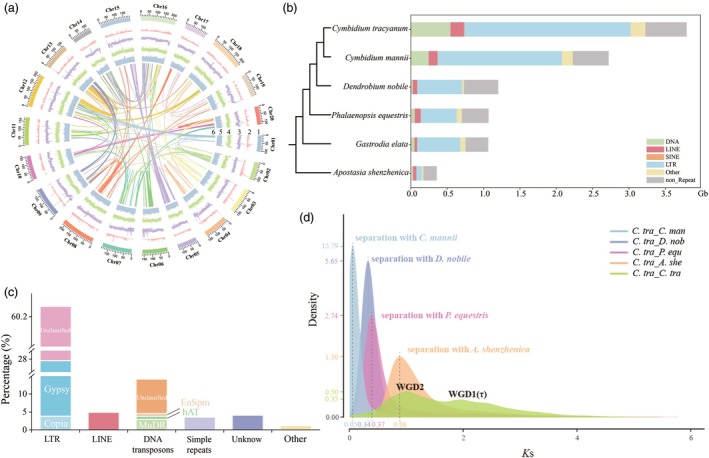
Genome features of *Cymbidium tracyanum* and the evolutionary history of whole‐genome duplication events. (a) Genomic features of *C. tracyanum*. The outer to inner circles represent: (1) chromosome length, with tick marks at 50 Mb intervals; (2–5) densities of genes, GC content, transposable elements (TEs) and long terminal repeats (LTRs), respectively, all plotted using a 100‐kb sliding window; (6) homologous regions among chromosomes, displayed with connecting lines colour‐coded according to chromosome number. (b) Comparison of repetitive sequences in orchids. (c) Relative contents of different repetitive element classes, with types constituting less than 0.5% of the genome not shown. (d) *K*s distribution and whole‐genome duplication (WGD) events in *C. tracyanum*. The *K*s distribution reveals two peaks: one at approximately 1.0 (WGD2), indicating the most recent WGD event shared by all orchids, and another at approximately 1.9 (WGD1), likely corresponding to the more ancient τ event shared by most monocots.

To elucidate the genetic mechanisms underlying floral scent, we first assembled the genome of *C. tracyanum* at the chromosome scale, laying a foundation for our research. The *C. tracyanum* genome assembly spans 3.79 Gb, comprising 16 288 contigs with a contig N50 of 1.66 Mb (Tables S1 and S2). Using chromatin interaction signals from Hi‐C data, 20 pseudochromosomes were constructed, with the longest pseudochromosome measuring 220.09 Mb and the shortest 106.15 Mb (Figures 1a, S1 and Table S2). The assembled genome size aligned with size estimates based on flow cytometry (3.95 Gb) and *k*‐mer frequency distribution (3.86 Gb) (Figure S2). Approximately 88.57% of the *C. tracyanum* genome consisted of repetitive elements, a proportion higher than that observed in currently sequenced orchids, such as *C. mannii* (82.8%) and *D. nobile* (61.07%) (Figure 1b). In *C. tracyanum*, most of the repetitive elements were transposable elements (TEs) (Table S3), with Long Terminal Repeats (LTRs) accounting for 60.27% of the genome, representing the largest proportion. Of these LTRs, *Gypsy* retrotransposons were the most prevalent, followed by *Copia* elements, accounting for 23.85% and 3.83% of the genome, respectively (Figures 1c and S3).

A total of 42 249 protein‐coding genes were annotated in the *C. tracyanum* genome with an average gene length of 7871 bp, coding sequence (CDS) length of 823 bp and an average of 4.13 exons per gene (Table S4). TrEMBL analysis revealed that 32 855 (77.77%) genes were functionally annotated, 24 735 of which encoded metabolic enzymes according to Kyoto Encyclopedia of Genes and Genomes (KEGG) pathways (Table S5). The annotation quality was assessed using Benchmarking Universal Single‐Copy Orthologs (BUSCO). A total of 1570 complete gene models out of 1614 (97.3%) were recovered, including 92.6% single‐copy genes and 4.7% duplicates, indicating high annotation quality and completeness (Table S6).

Whole‐genome duplication (WGD) events are prevalent among angiosperms and have significantly influenced agronomic or specialized phenotypic traits (Van de Peer *et al*., 2009). The distribution of synonymous substitutions per synonymous site (*K*s) of all paralogs in the *C. tracyanum* genome showed two distinct peaks in *K*s values (~1.0 and ~1.9) (Figure 1d), indicating two separate WGD events. The most recent WGD, inferred from the *K*s peak of approximately1.0 and consistent with other genome‐sequenced orchids, likely occurred before the divergence between *C. tracyanum* and *A. shenzhenica*, suggesting that this WGD event was shared by all extant orchids (Cai *et al*., 2014; Fan *et al*., 2023; Zhang *et al*., 2017; Xu *et al.*, 2022). Intragenomic collinearity analyses revealed that several chromosomal regions in *C. tracyanum* had one other syntenic region in the genome attributable to the orchid‐specific WGD, while some chromosomes, such as Chr08, showed up to three homologous syntenic regions, providing evidence for an even more ancient WGD (Figure S4), likely the more ancient τ event shared by most monocots (Jiao *et al*., 2014). Comparison with the chromosome‐level assembled genomes of *C. mannii* and *D. nobile* revealed excellent one‐to‐one correspondence (Figure S4), supporting the hypothesis that these species share the two WGD events observed in *C. tracyanum*.

### Phylogenomic analyses of *C. tracyanum*

**Figure 2:**
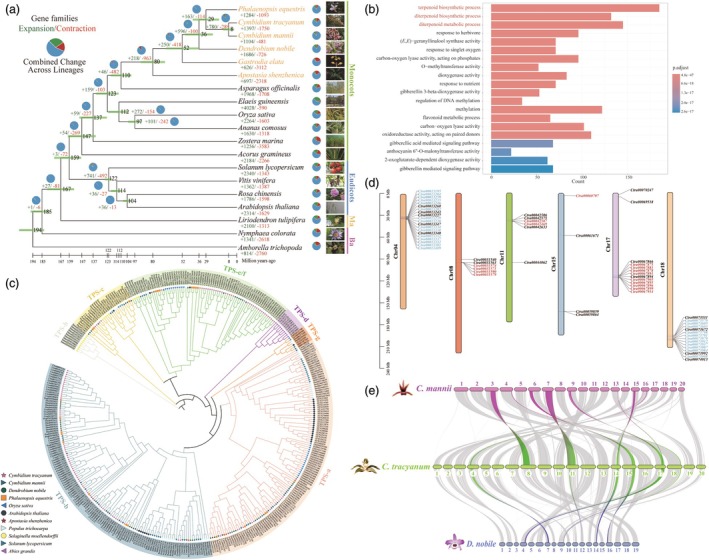
Phylogenomic analyses of *Cymbidium tracyanum*. (a) Phylogenetic tree of 19 plant species. Numbers represent divergence times at each node (Mya, million years ago), with green bars indicating 95% confidence intervals. Pie charts on branches show proportions of gene families undergoing expansion (green) and contraction (red). Numbers below pie charts correspond to the total number of expanded and contracted gene families, with colours matching pie charts. Ma and Ba represent Magnoliids and Basal angiosperms respectively. (b) Gene Ontology (GO) functional enrichment of expanded genes in the *C. tracyanum* genome (see Table S8 for details). (c) Phylogenetic tree of *TPS* genes from *Apostasia shenzhenica*, *Arabidopsis thaliana*, *Cymbidium mannii*, *C. tracyanum*, *Dendrobium nobile*, *Oryza sativa*, *Phalaenopsis equestris*, *Populus trichocarpa*, *Selaginella moellendorffii*, *Solanum lycopersicum* and *Abies grandis*. Detailed TPS proteins information is available in Table S10. (d) Schematic representation of the distribution of *TPS* genes in clusters on *C. tracyanum* chromosomes. Twenty *TPS* genes distributed across a stretch of 4.36 Mb on chromosome 4, 6 *TPS* genes distributed across a stretch of 4.23 Mb on chromosome 8, 5 *TPS* genes distributed across a stretch of 2.33 Mb on chromosome 11, 11 *TPS* genes distributed across a stretch of 4.43 Mb on chromosome 17, and 15 *TPS* genes occurred in 8.07‐Mb stretch on chromosome 18. Blue and red labels indicate segmental and tandem duplication events, respectively (see Table S11). (e) Synteny patterns between genomic regions from *C. tracyanum*, *C. mannii* and *D. nobile*. Highlighted segments denote collinear blocks containing *TPS* genes of *C. tracyanum*.

Orchids represent one of the most remarkable species radiations of flowering plants (Serna‐Sánchez *et al*., 2021). To elucidate the evolutionary history of *C. tracyanum*, we used 351 single‐copy orthologs across 19 plant species, selected based on their phylogenetic positions and floral scent phenotypes, to construct a phylogenetic tree and calculate divergence times (Table S7). As expected, *C. tracyanum* clustered with other orchids within the monocotyledonous clade, and the divergence times aligned with previous reports (Sun *et al*., 2021; Yang *et al*., 2021). As shown in Figure 2a, the divergence time between *C. tracyanum* and *C. mannii* was estimated to be 8 million years ago (Mya). The divergence time between *Cymbidium* and *Phalaenopsis* was estimated at 29 Mya, during the early Oligocene, and the origin of Orchidaceae was tracked back to approximately 110 Mya, during the mid‐Cretaceous.

The expansion or contraction of gene families has a profound role in driving phenotypic diversity and adaptive evolution in flowering plants (Lai *et al*., 2024). Based on our analysis of orchid phylogenetic relationships and divergence times, we identified gene families that significantly expanded or contracted at each ancestor node of 19 representative species (Figure 2a). Notably, a total of 1357 gene families (comprising 6861 genes) were specific to *C*. *tracyanum*. Additionally, 1397 gene families expanded, while 1750 families contracted. Intriguingly, Gene Ontology (GO) enrichment analysis revealed that the significantly expanded gene families were especially enriched in ‘terpenoid biosynthetic process’, ‘diterpenoid biosynthetic process’ and ‘diterpenoid metabolic process’ (Figure 2b and Table S8), which were apparently compatible with the strong aromatic quality specific to *C. tracyanum*. Terpenoid metabolites are not only essential for plant growth and development (e.g., gibberellins as phytohormones), but also serve as important intermediaries in various plant‐environment interactions (Tholl, 2006). This suggests that the terpene synthases (TPSs) may play a critical role in environmental adaptability of *C. tracyanum* by influencing the biosynthesis of floral volatiles.

### Significant expansion of the TPS gene family via tandem and segmental duplications

Plants have evolved diverse TPS gene families and subfamilies to synthesize specific terpenoid compounds, enabling them to interact effectively with both biotic and abiotic environments (Jiang *et al*., 2019). To understand this evolutionary diversification in the scented *C. tracyanum*, we identified and characterized 110 *TPS* genes—more than in any other of the sequenced orchid genome (Table S9). The TPS gene family is generally classified into seven clades (designated TPS‐a to TPS‐h) based on sequence similarity, functional assessment and gene structure (Chen *et al*., 2011). Hence, we constructed a phylogenetic tree incorporating *TPS* genes in orchids and other representative plant species to explore the potential functions of *TPSs* in *C. tracyanum* (Figure 2c). Orchid *TPS* genes were grouped into TPS‐a, ‐b, ‐c and ‐e/f subfamilies, excluding the TPS‐d subfamily, which is specific to gymnosperms, and the TPS‐h subfamily, which is unique to the lycophyte *Selaginella moellendorffii* (Chen *et al*., 2011). More than half of the *TPSs* in *C. tracyanum* belonged to the TPS‐b subfamily, which encodes monoterpene synthases (monoTPSs) in angiosperms, indicating that the diversification of monoTPSs likely contributed to the increase in the abundance of monoterpenes. The second most abundant *TPS* genes clustered into the TPS‐e/f subfamily, which encodes a variety of terpene synthases, including copalyl diphosphate synthases and kaurene synthases that are involved in the biosynthesis of gibberellic acid (Chen *et al*., 2011). Several *TPS* genes grouped into the TPS‐a subfamily, encoding sesquiterpene synthases (sesquiTPSs) in both monocots and dicots. Four *TPSs* of *C. tracyanum* were categorized into the TPS‐c subfamily, closely related to the TPS‐e/f subfamily and are conserved across land plants with diterpene synthase (diTPS) activity.

In many plant genomes, *TPS* genes frequently occur in tandem arrays, functioning as gene clusters (Chen *et al*., 2011; Martin *et al*., 2010). We analysed the distribution of *TPS* genes across their chromosomes in *C. tracyanum* (Figure 2d) and found that there were more than five *TPS* genes distributed in clusters on five chromosomes. These gene clusters were likely the result of tandem or segmental duplications (Figure 2d and Table S10). Notably, these clusters of genes belonged to the same TPS subfamily, respectively. In contrast, *TPS* genes distributed further apart on the same chromosome had significantly lower sequence similarity (Table S9).

Furthermore, we conducted a collinearity analysis between *C. tracyanum*, *C. mannii* and *D. nobile*, focusing on collinear blocks that contain *TPS* genes in *C. tracyanum* (Figure 2d). All chromosomes with clustered distributions of *TPS* genes in *C. tracyanum* (chromosomes 4, 8, 11, 17 and 18) exhibited collinear blocks with the other two species, which were also evident by the synteny dot plots (Figure S4).

### Profiles of floral volatiles in scented *C. tracyanum* and scentless *C. lowianum*

**Figure 3:**
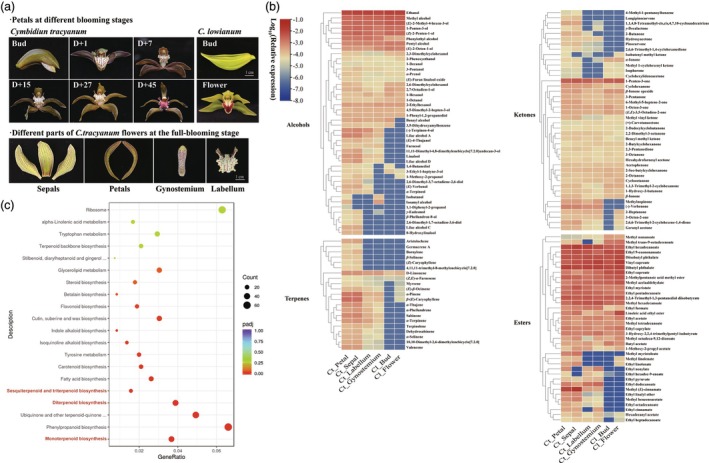
Profiles of floral volatiles in scented Cymbidium *tracyanum* and scentless *C. lowianum*. (a) Flowers at different developmental stages. Top: single flowers of *C*. *tracyanum* at six developmental stages (left), including the first day of flowering (D + 1), and 7 days (D + 7), 15 days (D + 15), 27 days (D + 27) and 45 days (D + 45) after flowering; Bud and full‐blooming flower (15 days after flowering) of *C. lowianum* (right); Below: different parts of *C*. *tracyanum* flowers at D + 15; Scale bars represent 1 cm; (b) Composition and content of volatile compounds detected in volatolomics of different parts of *C. tracyanum* flowers at the full‐blooming stage, and the petals of *C. lowianum* at the bud and full‐blooming stages (see Figure S5 and Table S12). (c) KEGG functional enrichment of differentially expressed genes in petals of *C. tracyanum* and *C. lowianum* at full‐blooming stage.

To identify components responsible for floral scent in *C. tracyanum*, we conducted volatolomics by GC–MS to measure volatile compounds in different parts of *C. tracyanum* flowers at the full‐blooming stage. These results were compared to those in *C. lowianum*, a species within the same genus that lacks scent (Figure 3a). We found that petals were the primary site of scent release. In total, 230 volatile compounds were identified (Table S11), including aldehydes (37), alcohols (44), terpenes (21), ketones (42), hydrocarbons (20), heterocyclic compounds (14), phenols (5), esters (37) and acids (10) (Figures 3b and S5).

Comparative analysis of volatile compounds in the petals of *C. tracyanum* and *C. lowianum* at the bud and full‐blooming stages identified several compounds with higher content in *C. tracyanum*. The vast majority of these compounds were terpenes, including monoterpenes such as α‐thujene, α‐phellandrene, sabinene, α‐terpinene, terpinolene and sesquiterpenes such as valencene and (*E*)‐β‐caryophyllene, as well as some alcohols (e.g., linalool, (‐)‐terpinen‐4‐ol), a few esters (e.g., methyl (*E*)‐cinnamate) and ketones (e.g., (‐)‐verbenone). These compounds were the significantly altered metabolites in the petals of full‐blooming flowers in *C. tracyanum* and *C. lowianum* (Table S12).

The emission patterns of terpenoids (terpenes and their oxygenated derivatives) were further characterized throughout flower development in *C. tracyanum*. We found that the production of terpenoids varied during flowering. Terpenoid emission, which was absent in floral buds, began on the day of anthesis (D + 1), increased rapidly during flower maturation (D + 15), peaked at full‐blooming stage (D + 27), and gradually decreased thereafter with flower decaying (Table S13). (*E*)‐β‐Caryophyllene accounted for the highest proportion of terpenoids. The OAV (odour activity value) of most terpenoids was greater than 1, indicating that they contributed to the floral scent of *C. tracyanum*. This was consistent with their differential expression detected by volatolomics (Table S12). In *C. lowianum* floral buds and blooming flowers, no terpenoids were detected (Figure S6). Additionally, volatile terpenoids were likely released directly from the epidermal cells of sepals and petals through a diffuse liberation mode, because no obvious osmophore was observed (Maffei, 2010; Vogel, 1983) (Figure S7).

### Comparative transcriptomics reveals key steps in terpenoid biosynthesis in *Cymbidium*

To explore the mechanisms that underlie differences in terpenoid biosynthesis between *C. tracyanum* and *C. lowianum*, we conducted a comparative analysis of their floral transcriptomes. To minimize tissue‐specific bias, transcriptomic data were generated from petals at various developmental stages. Samples from six developmental stages (Bud, D + 1, D + 7, D + 15, D + 27 and D + 45) of *C. tracyanum*, as well as floral bud and full‐blooming stages in *C. lowianum,* were examined (Table S14). KEGG enrichment analysis revealed that a total of 3862 differentially expressed genes in the petals of both orchids at full‐blooming stage were significantly enriched in pathways such as ‘Monoterpenoid biosynthesis’, ‘Sesquiterpenoid and triterpenoid biosynthesis’, and ‘Diterpenoid biosynthesis’ (Figure 3c). This finding was consistent with the observation that terpenoids were the main differential metabolites in floral volatiles. Similar enrichment patterns were also observed in the petals of *C. tracyanum* across different developmental stages, particularly during the transition from buds to the day of anthesis (Figure S8), when terpenoid emission began to increase rapidly (Table S13).

**Figure 4:**
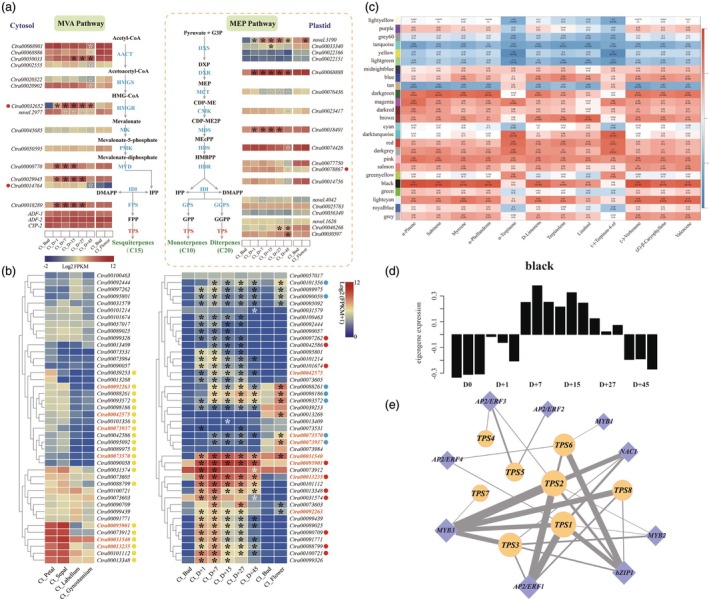
Gene expression profiles in terpenoid biosynthesis pathways and co‐expression analysis. (a) Comparative expression profiles of putative genes encoding enzymes involved in terpenoid biosynthesis in petals of *Cymbidium tracyanum* and *C. lowianum* at different developmental stages. Abbreviations for enzymes at each catalytic step are highlighted in blue and red. We set the threshold for differential gene expression as |log_2_(fold change)| ≥ 1 and padj ≤0.05. Genes meeting this threshold at different developmental stages are marked with asterisks at the corresponding stages, with solid asterisks indicating significant upregulation and hollow asterisks indicating significant downregulation. Red dots denote genes that are significantly highly expressed in the petals of *C. tracyanum* at the full‐blooming stage compared to *C. lowianum* (see Table S15). (b) Heatmaps depicting the expression patterns of *CtTPS* genes with FPKM >1 in different floral parts of *C. tracyanum* at the full‐blooming stage (left) and petals of *C*. *tracyanum* and *C. lowianum* at different developmental stages (right). Yellow dots represent genes that are significantly highly expressed in petals and sepals compared to the labellum and gynostemium in *C. tracyanum* at the full‐blooming stage. Red dots indicate genes that are significantly highly expressed in the petals of *C. tracyanum* compared to *C. lowianum*, while blue dots indicate significantly lower expression (see Table S16). The genes marked in orange represent *TPS* genes in the black module identified through the correlation analysis. (c) Matrix of module‐metabolite associations, with abscissa representing terpenoids and ordinate representing modules. Correlation coefficients and *P*‐values between modules and metabolites are shown at the row‐column intersections. (d) The expression pattern of genes within the black module is represented by its eigengene. (e) Co–expression network of nine transcription factors (CtAP2/ERF1‐4, CtMYB1‐3, CtNAC1 and CtbZIP1) and eight *CtTPS* genes in the black module. Diamonds represent transcription factors, and circles represent *CtTPS* genes. Node size indicates the importance of connectivity in the network, and edge thickness indicates the strength of the connectivity.

The putative genes encoding each step of the MVA and MEP pathways were further identified in both orchids (Table S15, Figures S9 and 4a). Additionally, the expression patterns of several key enzyme‐encoding genes across the floral developmental stages of the two orchids were validated through qRT‐PCR analysis (Figure S10). In the MVA pathway, HMG‐CoA synthase (HMGS) catalyses the production of HMG‐CoA from acetoacetyl‐CoA (AcAc‐CoA), a crucial step in both feedback regulation and stress responses (Tholl, 2015). Two *HMGS* genes were identified in *C. tracyanum* and *C. lowianum*, and neither exhibited significant changes across developmental stages in either species, nor did they show notable differences in the petals at the full‐blooming stage (Figures 4a and S10). HMG‐CoA reductase (HMGR) catalyses the irreversible formation of mevalonate from HMG‐CoA, which is considered a rate‐limiting step (Re *et al*., 1995). *C. tracyanum* possessed two *HMGR* genes: one showed an expression pattern consistent with flower development, peaking at the D + 7 before declining, whereas the other showed a continuous decline in expression from the bud stage to flower decay. In contrast, there was almost no difference in the expression levels of these two *HMGR* genes between the bud stage and the full‐blooming stage of *C. lowianum*, both of which remained at relatively low levels (Figures 4a, S10 and S11). In the MEP pathway, 1‐deoxy‐D‐xylulose 5‐phosphate (DXP) synthase (DXS) functions as an important regulatory and rate‐limiting enzyme to form DXP (Estévez *et al*., 2001). The four *DXS* genes in *C. tracyanum* belong to three clades, with enzymes in clade 2 mainly involved in the formation of plant secondary metabolites including terpenoids (Paetzold *et al*., 2010; Walter *et al*., 2002). This enzyme‐encoding gene (*novel.3190*) was also the only one that exhibited differential expression across developmental stages in both orchids. However, its expression levels were comparable at the full‐blooming stage in both species (Figures 4a, S10 and S12). Another key rate‐limiting enzyme, DXP reductoisomerase (DXR), which converts DXP to MEP by an intramolecular rearrangement, exhibited significantly differential expression compared to the bud stage during the entire flowering process of *C. tracyanum*. Whereas at different developmental stages, this gene expression in *C. lowianum* remained consistently high, comparable to that at the full‐blooming stage of *C. tracyanum* (Figures 4a and S10).

Transcriptome analysis of different floral parts of *C. tracyanum* flowers at the full‐blooming stage revealed that most genes (22/32) in MVA and MEP pathways, including the aforementioned rate‐limiting enzyme genes, exhibited significantly higher expression in petals and sepals compared to the labellum and gynostemium. This pattern was consistent with the profiles of terpenoids detected in the volatolomic analysis (Figure S13). As shown in Figure 4a, only one DXS enzyme‐encoding gene exhibited significant differential expression in the petals of *C. lowianum* between the full‐blooming and bud stages, while other genes maintained a stable expression pattern throughout the flowering process of *C. lowianum*. Additionally, across the entire pathway, only three genes (*Ctra00032652*, *Ctra00014764* and *Ctra00078867*) showed significantly higher expression in the petals of *C. tracyanum* at the full‐bloom stage compared to *C. lowianum*. This suggested that the differences in terpenoid content among different floral parts of *C. tracyanum* might be primarily due to variations in substrate synthesis supplied to TPSs. In contrast, the differences in terpenoid content between the petals of the two orchids were less influenced by this factor but were more likely driven by the substrate‐specific catalytic activity of downstream TPSs, which further contributed to variations in both the types and amounts of terpenoids.

We also analysed the expression levels of 110 *TPS* genes identified in *C. tracyanum* (Figure S14), focusing on 42 genes with an average FPKM >1 in different floral parts at the full‐blooming stage. Among them, half of the genes exhibited significantly higher expression levels in petals and sepals compared to the labellum and gynostemium. Moreover, we found that the expression of several *TPS* genes in petals changed with floral developmental stages in *C*. *tracyanum* and *C. lowianum* (Table S16 and Figure 4b).

### Co‐expression network related to terpenoid biosynthesis in *C. tracyanum* identifies candidate genes involved

To further clarify the key *TPS* genes and regulatory elements responsible for terpenoid biosynthesis in *C. tracyanum*, we conducted a weighted correlation network analysis (WGCNA) by combining the floral transcriptome data from different developmental stages and corresponding terpenoid content measurements (Figure S15 and Table S13). The analysis revealed correlations between 25 modules and 12 characteristic terpenoids detected throughout flower development in *C. tracyanum* (Figure 4c). Modules with larger correlation coefficients and lower *P*‐values were highly correlated with specific phenotypes. The black module exhibited the strongest correlation with the majority of terpenoids, including α‐pinene, sabinene, myrcene, D‐limonene, (‐)‐verbenone, (*E*)‐β‐caryophyllene and valencene. The expression of this module, represented by its eigengene, increased during the blooming process, peaked at the full‐blooming stage and then declined (Figure 4d). Eight *TPS* genes (*Ctra00013235*, *Ctra00031540*, *Ctra00042575*, *Ctra00073570*, *Ctra00073937*, *Ctra00092263*, *Ctra00094506* and *Ctra00095901*) were identified (designated as *CtTPS1*–*CtTPS8*). These genes were expressed at relatively higher levels in the petals and sepals of full‐blooming flowers (Figure 4b).

The emission of terpenoids is not only affected by TPS, but also regulated by various *cis*‐elements at the transcriptional level. Here, a total of 642 genes were annotated as bHLH, bZIP, EIL, MYB, NAC, WRKY and AP2/ERF transcription factors. Among these, nine transcription factors were identified as potential regulators of terpenoid synthesis, based on their connectivity with eight *CtTPS* genes in the black module. These TFs were nominated as CtAP2/ERF1–4, CtMYB1–3, CtNAC1 and CtbZIP1 (Table S17). The regulatory network mediated by these TFs and *CtTPS* genes was shown in Figure 4e. Notably, CtAP2/ERF1, CtbZIP1, CtNAC1 and CtMYB2,3, displayed high co‐expression with *CtTPS1, 2, 3, 8*, suggesting their roles as hub genes in the network.

**Figure 5:**
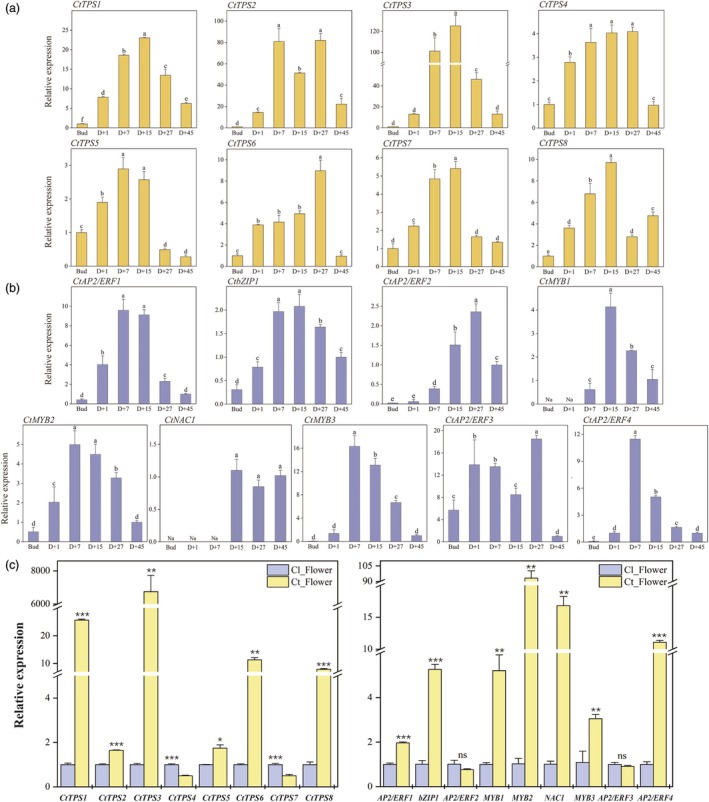
Gene expression levels of CtTPSs and CtTFs at different developmental stages analysed by qRT‐PCR. (a, b) Expression levels of *CtTPS* genes (a) and *CtTF* genes (b) at different developmental stages in the petals of *C. tracyanum*. *CtCYP2* was used as an internal control. Different letters represent significant differences between stages, calculated through one‐way ANOVA (*P* < 0.05). Error bars indicate the standard deviation (SD) of three replicates. (c) Expression levels of eight structural genes (left) and nine candidate TFs (right) in the petals of scentless *C. lowianum* and scented *C. tracyanum* (at the D + 15 stage). Statistical analysis of the expression levels of genes between the two orchids was performed by using Student's *t*‐test (*P < 0.05, **P < 0.01 and ***P < 0.001). Expression levels are normalized to *CtCYP2*. Data are represented as means ± SD from three replicates. Primers are listed in Table S21.

To validate the expression patterns of genes identified through co‐expression analysis, the transcript abundances of eight selected *CtTPSs* and nine *CtTFs* in the petals of *C. tracyanum* at various floral developmental stages were analysed by qRT‐PCR. Notably, *CtTPS5* was expressed at lower levels at D + 27 than at the bud stage, while the expression of other structural genes involved in terpenoid biosynthesis generally increased, peaking during flowering before declining. This pattern mirrored the trend of terpenoid emissions. Although the expression levels of *CtTPS2* and *CtTPS8* fluctuated during floral development, they consistently showed significantly higher expression levels at all blooming stages compared to the bud stage (Figure 5a). The expression of the 9 *TFs* either peaked on D + 15 (*CtbZIP1*, *CtMYB2* and *CtNAC1*), D + 27 (*CtAP2/ERF2*, *3*) or D + 7 (*CtAP2/ERF1*, *4* and *CtMYB2*, *3*), preceding the expression of the structural genes (Figure 5b).

We also compared the gene expression across different floral parts of *C. tracyanum* at the full‐blooming stage (D + 15). All *CtTPS* genes showed significantly higher expression levels in petal and sepal compared to labellum and gynostemium, with *CtTPS1* expression approximately 50‐fold higher in petal and sepal. *CtTPS5* exhibited the least variation in expression across different flower parts, lacking clear tissue specificity (Figure S16). For transcription factors, *CtbZIP1*, *CtMYB1*, *2*, *3* and *CtAP2/ERF4* were highly expressed in petal and sepal. *CtAP2/ERF1* showed comparable expression levels in petal, sepal and gynostemium, while *CtAP2/ERF3* exhibited the highest expression in gynostemium. *CtNAC1* had similar expression levels in the petal and gynostemium but was significantly higher than in the sepal (Figure S17). Structural genes and transcription factors involved in terpenoid biosynthesis in *C. tracyanum* are expected to be expressed at higher levels in scented *C. tracyanum* than in scentless *C. lowianum*. We found that *CtTPS1*, *2*, *3*, *5*, *6* and *8* were highly expressed in *C. tracyanum* but were rarely detectable in scentless *C. lowianum*. However, *CtTPS4* and *CtTPS7* showed higher expression in *C. lowianum* than in *C. tracyanum*. The expression of *CtAP2/ERF2*, *3* did not differ significantly between the two orchids, but *CtAP2/ERF1*, *4*, *CtbZIP1*, *CtMYB1*, *2*, *3* and *CtNAC1* were expressed at remarkably higher levels in scented *C. tracyanum* than in scentless *C. lowianum* (Figure 5c).

Based on these findings, we identified four *CtTPSs* (*CtTPS1*, *2*, *3*, *8*) and six CtTFs (CtAP2/ERF1, 4, CtbZIP1 and CtMYB1, 2, 3) as key structural genes and regulators, showing specifically high expression in the petal and sepal of *C. tracyanum*. Their expression patterns were consistent with terpenoid emissions during flower development.

### Versatile and diverse functions of *CtTPS* genes

**Figure 6:**
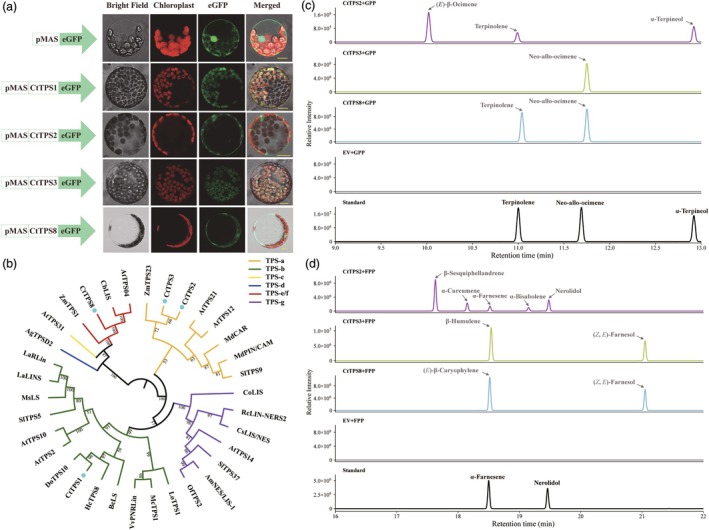
Functional characterization of the enzymes encoded by *CtTPS* genes. (a) Subcellular localization of free eGFP and four CtTPS‐eGFP fusions in *Nicotiana benthamiana* leaf protoplasts. eGFP fluorescence was detected in the green channel, while chlorophyll autofluorescence was detected in the red channel; Merged, brightfield image combined with green and red channels; scale bar indicates 10 μm. (b) Phylogenetic analysis of TPS proteins from *Cymbidium tracyanum* (CtTPS1, 2, 3, 8) and other plants using the maximum likelihood method. The CtTPS proteins in this study are highlighted by circles. The TPS‐a, TPS‐b, TPS‐c, TPS‐d, TPS‐e/f and TPS‐g clades are highlighted with shaded lines. Plant species are as follows: *Arabidopsis thaliana* (At); *Antirrhinum majus* (Am); *Abies grandis* (Ag); *Backhousia citriodora* (Bc); *Clarkia breweri* (Cb); *Cinnamomum osmophloeum* (Co); *Camellia sinensis* (Cs); *Dendrobium officinale* (Do); *Hedychium coronarium* (Hc); *Lavandula angustifolia* (La); *Lilium* hybrid cultivar (Lo); *Magnolia champaca* (Mc); *Malus domestica* (Md); *Mentha spicata* (Ms); *Osmanthus fragrans* (Of); *Rosa chinensis* (Rc); *Solanum lycopersicum* (Sl); *Vitis vinifera* (Vv); *Zea mays* (Zm). Detailed information on TPS proteins is provided in Table S18. (c, d) Identification of enzymatic products of recombinant CtTPS proteins using geranyl diphosphate (GPP)/farnesyl diphosphate (FPP) as substrates. Volatile terpenoids were analysed by GC–MS and extracts from the *E*. *coli* expression system containing empty vector were used as controls. Arrows indicate compounds identified based on database matching, while “Standard” refers to the detection results of standard samples under the same conditions (Table S19).

Phylogenetic analysis was conducted to infer the potential catalytic functions of the four CtTPS proteins by comparing them with other functionally validated plant TPSs (Figure 6b and Table S18). CtTPS2 and CtTPS3 clustered into the TPS‐a subfamily, which represents sesquiTPSs in monocots and dicots. CtTPS1 was classified into the TPS‐b subfamily, representing monoTPSs in angiosperms. Notably, DoTPS10, closely related to CtTPS1, is one of the few TPSs in orchids that have been identified to catalyse linalool synthesis *in vitro* (Yu *et al*., 2020). Additionally, CtTPS8 belongs to the subfamily TPS‐e/f that has enzyme activities of monoTPSs, sesquiTPSs and diTPSs in vascular plants (Chen *et al*., 2011). Subcellular localization analysis was performed by fusing the full‐length sequences with the eGFP protein. The results revealed that CtTPS1 and CtTPS3 were localized in the plastids, whereas CtTPS2 and CtTPS8 were distributed in the cytoplasm (Figure 6a). However, the subcellular localization of terpene synthases and their functional sites is not entirely consistent (Bao *et al*., 2023; Conart *et al*., 2023).

Previous studies have suggested that *TPS* genes evolve rapidly, and even with high sequence similarity, their catalytic products can vary considerably (Jia *et al*., 2022). To directly confirm the enzymatic properties of these CtTPS proteins, we performed *in vitro* assays using GPP or FPP as substrates. Recombinant CtTPS proteins were expressed heterologously in *E. coli* for biochemical analysis (Figure S18). As expected, no products were detected in reactions where heat‐inactivated recombinant proteins were added to reaction mixtures supplemented with both substrates. Thus, only crude protein extracts from the *E*. *coli* expression system containing empty vector were used as controls. Upon incubation with GPP as a substrate, preliminary identification based on comparison with the NIST database indicated that CtTPS2 produced (*E*)‐β‐ocimene, α‐terpineol and terpinolene, all of which were detected in the *C. tracyanum* flowers. CtTPS3 catalysed the formation of neo‐allo‐ocimene, whereas CtTPS8 not only catalysed neo‐allo‐ocimene but also produced comparable amounts of terpinolene. Furthermore, the production of neo‐allo‐ocimene, terpinolene and α‐terpineol was confirmed using authentic standards (Figure 6c and Table S19). The catalytic activity of recombinant CtTPS proteins was also performed using FPP as a substrate. Preliminary comparison results indicated that CtTPS2 could catalyse the production of a variety of sesquiterpenes, including β‐sesquiphellandrene, α‐curcumene, α‐farnesene, α‐bisabolene and nerolidol, demonstrating the diverse catalytic functions of CtTPS2. CtTPS3 could produce β‐humulene and (*Z*, *E*)‐farnesol. The main product of CtTPS8 was (*E*)‐β‐caryophyllene, which was also the most abundant compound during the flowering process of *C. tracyanum*. The formation of α‐farnesene and nerolidol was further confirmed using authentic standards (Figure 6d and Table S19). The *in vitro* catalytic assays of these recombinant CtTPS proteins partially explain the biosynthesis of certain floral scent compounds in *C. tracyanum*. It was also evident that CtTPS2, CtTPS3 and CtTPS8 are all bifunctional enzymes *in vitro*, while their actual product profiles in planta could be influenced by substrate availability and enzyme localization.

### *CtTPS* genes directly regulated by selected TFs

**Figure 7:**
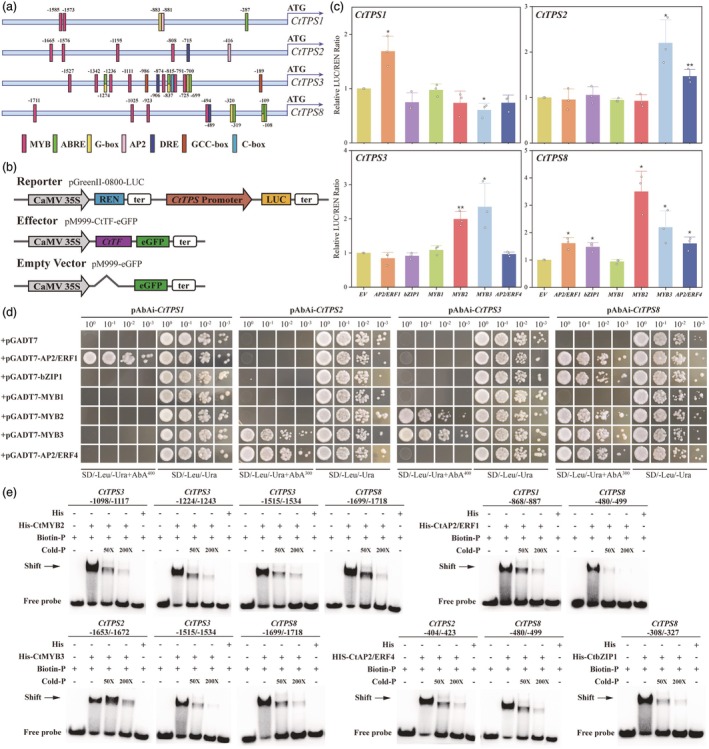
Confirmation of *CtTPS* genes directly regulated by TFs. (a) Schematic diagram of *cis*‐elements in the promoter region of four *CtTPS* genes. MYB: MYB structural domain; ABRE: ABA response elements; G‐box: CACGTG; AP2: AP2 domain; DRE: GCCGAC; GCC‐box: GCCGCC; C‐box: GACGTC. (b) Schematic diagram of vector construct. Reporter vector: pGreenII‐0800‐LUC‐proCtTPS; effector vector: pM999‐CtTF‐eGFP; empty vector: pM999‐eGFP. (c) Activation of six CtTFs on four *CtTPS* genes was assessed by dual‐luciferase assays in tobacco leaf protoplasts. The empty pM999‐eGFP vector served as a negative control. Error bars represent the SD of three replicates. Asterisks denote significance levels calculated by Student's *t*‐test compared with negative control (*P < 0.05, **P < 0.01 and ***P < 0.001). (d) Yeast one‐hybrid assays demonstrating that CtTFs specifically bind to the promoters of different *CtTPS* genes. The empty vector pGADT7 vector served as the negative control. SD medium is the Ura and Leu double deficient medium. Superscripted numbers indicate the concentration of aureobasidin A used for screening. (e) Electrophoretic mobility shift assays (EMSAs) showing direct binding of His‐CtTFs to the promoter of *CtTPS* genes. Each element, along with its upstream and downstream flanking sequences, was biotin‐labelled and used as a probe. A shifted band (indicated by an arrow) represents the protein‐DNA complex. Competition assays were performed using an excess of unlabeled probe (50‐fold and 200‐fold) to confirm binding specificity. A His‐tagged control protein was used as a negative control. Experiments were repeated at least twice with consistent results.

After elucidating the functions of CtTPS proteins, we sought to understand the regulatory mechanisms by which transcription factors control these terpene synthases. Initial subcellular localization analysis confirmed that all six transcription factors were localized to the cell nucleus (Figure S19). We then cloned the promoter sequences (~2 kb upstream) of the four *CtTPS* genes and identified several *cis*‐elements within these regions, indicating potential binding sites for candidate TFs (CtAP2/ERF1, 4, CtbZIP1 and CtMYB1, 2, 3). These *cis*‐elements mainly included the AP2/ERF transcription factor‐specific binding GCC‐box, AP2 and DRE/CRT, the bZIP transcription factor‐specific binding G‐box, C‐box and ABRE, and binding sites for MYB transcription factors (Figure 7a).

Subsequently, we constructed promoter activation dual‐luciferase reporter assay vectors with the coding sequences of the TFs under the control of the CaMV 35S promoter. Promoter fragments of *CtTPSs* were integrated into the pGreenII‐0800‐LUC vectors to perform the promoter activation experiment in tobacco leaf protoplasts (Figure 7b). As shown in Figure 7c, based on the expression ratio of LUC/REN compared to controls (empty vector without TF), we found that CtAP2/ERF1 activated the promoters of *CtTPS1* and *CtTPS8*, CtbZIP1 only activated the promoter of *CtTPS8*, CtMYB2 activated the promoters of *CtTPS3* and *CtTPS8*, and CtAP2/ERF4 activated the promoters of *CtTPS2* and *CtTPS8*. Additionally, CtMYB3 exhibited activation of the promoters of *CtTPS2*, *3* and *8*. Further examination of the activation of the *CtTPSs* promoter regions by selected TFs using yeast one‐hybrid assays yielded consistent binding results (Figure 7d). In addition, electrophoretic mobility shift assays (EMSA) were conducted to provide direct biochemical evidence of TF binding, reinforcing their regulatory roles in *CtTPS* gene expression (Figure S20 and Table S21). As shown in Figure 7e, CtAP2/ERF1 was able to directly bind to the AP2 domain in the *CtTPS1* promoter region and the DRE element in the promoter of *CtTPS8*. Similarly, CtAP2/ERF4 was directly bound to the AP2 domain in the *CtTPS2* promoter region and the DRE element in the promoter of *CtTPS8*. The activation of *CtTPS8* by CtbZIP1 was attributed to its binding to the G‐box and ABA response element. CtMYB2 and CtMYB3 bound to the MYB sites in their respective gene promoter regions, with CtMYB2 specifically interacting with three MYB sites in the *CtTPS3* promoter. It was worth noting that the results of transcription factor activation were partially consistent with our putative co‐expression network (Figure 4e), with some TFs exhibiting regulatory roles as predicted. These findings verified that the selected TFs regulate the expression of terpenoid biosynthesis‐related genes.

## Discussion

Here, we employed multi‐omics, molecular biology and phytochemistry to elucidate the mechanisms underlying the biosynthesis and regulation of floral scent in *C. tracyanum*. Comparative genomic analysis through high‐quality chromosome‐level genome assembly revealed that the significantly expanded gene families in *C. tracyanum* were primarily enriched in pathways related to terpenoid biosynthesis and metabolism. Our integration of comparative transcriptome and metabolome analyses identified several essential terpene synthases (i.e. *CtTPS1*, *CtTPS*2, *CtTPS3* and *CtTPS8*). We verified the catalytic function of CtTPSs and identified transcription factors that regulate *CtTPS* gene expression, including CtAP2/ERF1, CtbZIP1, CtMYB2, CtMYB3 and CtAP2/ERF4.

### The contribution of significantly expanded *TPS* genes in *C. tracyanum* to volatile terpenoid biosynthesis and its implications for adaptive evolution

Our comparative genomic analyses, based on the newly released *C. tracyanum* genome, revealed that significantly expanded gene families in *C. tracyanum* are predominantly enriched in pathways related to terpenoid biosynthesis and metabolism. This finding is consistent with its strong floral scent phenotype, suggesting that terpenoids have played a crucial role in the environmental adaptability of *C. tracyanum* throughout its evolutionary history. Our finding that TPS‐a and TPS‐b subfamily genes have undergone extensive expansion in *C. tracyanum* is consistent with previous studies, which indicate that their expansion contributes to the diversification and accumulation of monoterpenes and sesquiterpenes (Huang *et al*., 2021; Shang *et al*., 2020; Zhang *et al*., 2021). GO enrichment analysis of the contracted gene families revealed significant enrichment in pathways related to transcription factor or transcription regulator activity, stress responses, as well as pathways associated with plant organ morphogenesis. This might also reflect the strategies of *C. tracyanum* at the genomic level in pathway regulation and adaptation to specific life forms during evolution (Figure S21 and Table S20).

Previous studies have shown that the TPS family has undergone several expansions due to gene duplications during plant evolution, leading to structural remodelling, differential expression, subcellular segregation and neofunctionalization (Jia *et al*., 2022; Karunanithi and Zerbe, 2019). Our results revealed that *C. tracyanum* has experienced two major duplication events in its evolutionary history, with an ancient event shared by monocots and a more recent event shared by all species within the Orchidaceae (Figure 1d). Yet, the number of TPS gene family members in *C. tracyanum* surpasses that of any other sequenced orchid (Table S10), likely due to a combination of tandem and segmental duplications (Table S11). Further research is needed to confirm the existence of functional redundancy or divergence among *TPS* genes in *C. tracyanum* from different sources, particularly the potential role of those distributed in clusters on chromosomes (Figure S9).

Interestingly, some *TPS* genes were scarcely expressed in *C. tracyanum* flowers (Figure S14), suggesting potential functional divergence or even neofunctionalization. Beyond their role as floral volatiles, terpenoids also participate in plant growth and development as essential components and regulatory substances (Christianson, 2006; Pichersky *et al*., 2006; Tholl, 2006). In addition, volatile terpenoids may compete with other substances for metabolic flux. For instance, floral colours, which are often discussed alongside floral scents, are biochemically connected with VOCs despite their distinct biosynthetic pathways. They exhibit synergistic interactions during flower development and respond to biotic and abiotic stresses (Li *et al*., 2025). Similarly, the differentially expressed genes in the petals of *C. tracyanum* and *C. lowianum* at the full‐blooming stage were significantly enriched in the phenylpropanoid biosynthesis pathway (Figure 3c). Compared to the lack of corresponding differences in volatolomics, this phenomenon was more likely attributed to the differences in flower colour between the two species (Figure 3a). It also reflects the diverse roles of phenylpropanoid pathway products in the structural, physiological and ecological functions of the plants (Vogt, 2010; Wang *et al*., 2014).

### Mechanisms of biosynthesis and regulation of volatile terpenoids in *C. tracyanum*

The floral terpenoids in *C. tracyanum* exhibited significant tissue and spatiotemporal specificity throughout the developmental stages (Figures 3b, S5 and Table S13). The highest levels of VOCs were detected in the petal and sepal, whereas the lowest levels were detected in the labellum and gynostemium, a pattern that may avoid damaging critical pollination structures. During flower development, VOC content first increased and then decreased. This pattern has been observed in other plants and reflects an initial attraction of pollinators followed by the avoidance of further damage to pollinated flowers (Cancino and Damon, 2007). However, this speculation needs to be further analysed by examining changes in floral volatiles before and after pollination and orchestrating specific pollinator attraction experiments.

Terpenoids are one of the most diverse classes of plant metabolites, with significant physiological and ecological functions. TPSs, as pivotal enzymes that initiate terpenoid biosynthesis, have drawn considerable attention. Although the identification of TPSs across a broad range of plant lineages, including angiosperms, gymnosperms, bryophytes, lycophytes and ferns, has extended our understanding of the origins and evolution of TPSs (Jia *et al*., 2022), model plants, such as *Arabidopsis thaliana* and *Oryza sativa*, have served as paradigms for in‐depth studies of the spatial organization and temporal regulation of TPSs in relation to various biological functions (Chen *et al*., 2014; Huang *et al*., 2011). Few studies have examined the regulation of terpenoid biosynthesis in orchids, although one report on *Phalaenopsis* demonstrated that PbbHLH4 regulates floral monoterpene biosynthesis (Chuang *et al*., 2018).

Here, we examined the expression patterns of *CtTPSs* at different developmental stages and in different parts of *C. tracyanum* flowers. We also used co‐expression analysis to screen for transcription factors that may regulate *CtTPS* genes (Figure 4e). Our analysis of *cis*‐element binding sites in the promoter region of the *CtTPS* genes (Figure 7a) and the subsequent validation experiments through dual‐luciferase reporter assays, yeast one‐hybrid experiments and EMSA assays (Figure 7c–e) identified several TFs that activate the promoter regions of multiple *CtTPS* genes. Specifically, CtAP2/ERF1 activated the promoters of *CtTPS1* and *CtTPS8*; CtMYB2 activated the promoters of *CtTPS3* and *CtTPS8*, and CtAP2/ERF4 activated the promoters of *CtTPS2* and *CtTPS8*. CtMYB3 activated the promoters of *CtTPS2, CtTPS3* and *CtTPS8*. This suggested that CtMYB3 is likely a key transcription factor involved in regulating the floral scent volatiles of *C. tracyanum* and warrants further investigation. These findings showed that *CtTPS* promoters could be bound by multiple transcription factors. Meanwhile, our study also provided a solid foundation for the functional analysis of other terpene synthases in *C. tracyanum*. It remains unclear whether these transcription factors simultaneously activate and/or repress promoter regions and how their combined actions regulate the synthesis of terpenoids in *C. tracyanum*. Currently, the lack of a suitable genetic transformation system limits our ability to directly verify the regulatory effects of transcription factors on *CtTPSs* in *C. tracyanum*.

### Our study provides a solid reference for in‐depth exploration of floral scent and other unique adaptive traits in orchids

Since Darwin's publication of *Fertilisation of Orchids* in 1862, orchids have garnered significant attention from evolutionary biologists. The genome of *C. tracyanum* offers new avenues to delve into the genomic basis of the physiological characteristics in orchids. Although studies on orchid floral scent are common, they predominantly focus on the detection of volatile compounds or the identification of genes in related pathways. Comprehensive studies that encompass the spatiotemporal changes of volatile compounds, genome assembly and comparative analysis, expression patterns of structural genes involved in terpenoid biosynthesis, as well as the verification of their catalytic functions and transcriptional regulation, are quite rare in orchids. However, many aspects of terpenoid biosynthesis in orchids still warrant in‐depth exploration.

First, attention should be given to the biosynthesis of several other floral VOCs, such as those in the benzenoid/phenylpropanoid biosynthesis pathway and the biosynthetic pathway of volatiles derived from fatty acids. Although these VOCs are not as abundant as terpenoids, they may still impact the floral scent of *C. tracyanum*. For example, in *Caladenia plicata*, a new floral volatile constituent, 2‐hydroxy‐6‐methylacetophenone, has been shown to cooperate with the commonly reported (*S*)‐β‐citronellol in the attraction of pollinators (Xu *et al*., 2017). Secondly, the focus should be placed on the role of floral VOCs as signalling molecules that attract pollinators and facilitate communication between plants and the environment, especially the conversion between volatile and non‐volatile substances, such as the identification of subsequent modification enzymes. Modification of TPS products by oxidation, peroxidation, methylation, acylation, or cleavage changes their physical properties and may alter their biological activities (Chen *et al*., 2011). Furthermore, in some plants, such as *Freesia* (Bao *et al*., 2023), the functions of natural allelic variants of TPS among different varieties within the same genus have been identified. In rose, differences in the content of citronellol in different species have been shown to be due to *NUDX1‐1a* copy numbers, and the evolution of biosynthetic pathways of terpene scent compounds within the genus has been elucidated (Shang *et al*., 2024). For *Cymbidium* species with such rich floral scent resources, understanding the differences in the biosynthesis of floral scent compounds among different varieties, as well as the evolutionary mechanisms within this genus, is crucial for guiding breeding practices and understanding their adaptability to the environment.

## Conclusions

In the present study, we identified terpenoids as the primary contributors to floral scent in *C. tracyanum* and uncovered a significant expansion of gene families related to terpenoid biosynthesis and metabolism in its genome. We further validated the roles of several *CtTPS* genes in the biosynthesis of volatile terpenoids in *C. tracyanum* flowers and explored the potential regulatory networks involving these genes and associated transcription factors. The integration of multi‐omics data significantly advances our understanding of floral scent biosynthesis in orchids. Moreover, the availability of a newly generated genome of *C. tracyanum* provides a valuable resource for comparative genomics studies, shedding light on the diversity of ornamental traits and the evolutionary mechanisms of adaptive traits in orchids at the genomic level. These findings are of great importance for breeding fragrant orchids and understanding the adaptation and evolution of orchids.

## Materials and methods

### Plant materials and growth conditions

All plants used in this study were cultivated in a greenhouse at Kunming Institute of Botany, Kunming, China. The greenhouse conditions were maintained at an air temperature of 18–24 °C, with relative humidity (RH) of 50%–70% and exposure to 30% of full sunlight. For *C*. *tracyanum*, flower samples were collected at six developmental stages: buds (Bud), the first day of flowering (D + 1), and 7 days (D + 7), 15 days (D + 15), 27 days (D + 27) and 45 days (D + 45) after flowering. A portion of fresh flowers from six developmental stages was used for the measurement of VOCs, and petals at each stage were collected for RNA extraction. In addition, the flowers at D + 15 were further divided into four parts: petal, sepal, labellum and gynostemium. Each part was collected separately for tissue sample subsets. For *C. lowianum*, petals were collected from buds and the full‐blooming stage (15 days after flowering). All samples were used for volatolomics detection and RNA extraction. Samples were immediately frozen in liquid nitrogen and stored at −80 °C until required.

### Genomic DNA preparation and sequencing

Healthy leaves from the same *C. tracyanum* individual were collected, immediately transferred to liquid nitrogen, and stored at −80 °C until DNA extraction. High‐molecular‐weight genomic DNA used for both short‐read and long‐read DNA sequencing was extracted using the cetyltrimethylammonium bromide (CTAB) method. The quality of the DNA was checked using 0.5% agarose gel electrophoresis, and the concentration of the DNA was determined by Qubit fluorimeter (Invitrogen, MA, USA). Genomic DNA sequencing was performed at Wuhan BGI Technology Co., Ltd. on both HiSeq X Ten (Illumina, CA, USA) and Sequel (Pacific Biosciences, CA, USA) platforms. Two Illumina libraries produced 462 Gb of short reads, and 43 SMRTbell libraries produced 403 Gb of long reads (Table S1).

### Genome assembly and evaluation

The genome size of *C. tracyanum* was estimated by analysing *k*‐mer (17‐mer) frequencies in whole‐genome sequencing data produced previously, and further experimentally validated by flow cytometry. Contigs were assembled using FALCON v0.5 (Chin *et al*., 2016), followed by polishing and error correction with PacBio and Illumina sequencing reads by using *Arrow* (SMRTlink release_6.0.0.47841) and Pilon v1.22 (Walker *et al*., 2014). The TrimDup module in Rabbit was used to remove redundant and heterozygous sequences (Chen *et al*., 2020). The completeness of the assembled *C*. *tracyanum* genome was assessed using BUSCOs v5.5.0 (Manni *et al*., 2021) against the embryophyta_odb10 dataset.

### Pseudochromosome construction with Hi‐C data

To construct pseudochromosomes, *C. tracyanum* leaf cells were treated with formaldehyde to fix the cross‐linked complexes. The cross‐linked DNA was then digested using the restriction enzyme *Mbo*I. The sticky ends were labelled with biotin and ligated to form circular chimeric molecule. Purified and fragmented DNA was selected for library construction. Four libraries were sequenced on Illumina HiSeq X Ten and generated 544 Gb of paired‐end reads. Uniquely mapped read pairs were analysed using HiC‐Pro (Servant *et al*., 2015). The assembled contigs were anchored and ordered into a chromosome‐level assembly using Juicer v1.5 (Durand *et al*., 2016b) and 3D‐DNA v180922 (Dudchenko *et al*., 2017). Finally, we reviewed and refined the assembly with Juicebox v1.11.08 (Durand *et al*., 2016a).

### Genome annotation

The annotation of repetitive sequences, including tandem repeats and TEs, was performed prior to the gene annotation of C. *tracyanum*. Tandem repeats were identified by Tandem Repeats Finder v4.09 (Benson, 1999). Transposable elements (TEs) were identified by a combination of homologue and *de novo* approaches. RepeatMasker v4.0.7 and RepeatProteinMask were used to annotate the TEs in the genome (Tarailo‐Graovac and Chen, 2009). The *de novo* repeat library was predicted by RepeatModeler v1.0.4 and LTR_FINDER v1.0.6 (Xu and Wang, 2007). For *de novo* gene model prediction, AUGUSTUS v3.2.1 (Stanke *et al*., 2006) and SNAP (Johnson *et al*., 2008) were employed. For homology‐based annotation, protein sequences from five sequenced plants, *Arabidopsis thaliana* (Cheng *et al*., 2017), *Oryza sativa* (Ouyang *et al*., 2007), *Asparagus officinalis* (Harkess *et al*., 2017), *Phalaenopsis aphrodite* (Chao *et al*., 2018) and *Gastrodia elata* (Yuan *et al*., 2018) were downloaded and mapped onto the *C. tracyanum* genome using TBLASTN (Camacho *et al*., 2009) followed by inferring the exon‐intron boundaries using Exonerate v2.2.0 (Slater and Birney, 2005). Eight healthy tissues (root, root tip, stem, leaf, bract, flower bud, opening flower and fruit) were collected for RNA extraction and sequences from eight cDNA libraries were used for genome annotation on the HiSeq 2500 platform. Mixed RNA from these eight tissues was used for IsoSeq sequencing. cDNA products were amplified using KAPA HiFi PCR kits (KAPA Biosystems, Cape Town, South Africa), followed by purification using the SMRTbell Template Prep Kit (Pacific Biosciences, CA, USA). Libraries were sequenced on the PacBio Sequel II platform (Table S1). Finally, *de novo*, transcriptome‐based and homology‐based approaches were combined to predict gene function by Maker v2.31.8 (Holt and Yandell, 2011) Subsequently, the Swiss‐Prot, TrEMBL (Boeckmann, 2003), Kyoto Encyclopedia of Genes and Genomes (Ogata *et al*., 1999), InterPro (Zdobnov and Apweiler, 2001) and Gene Ontology (Ashburner *et al*., 2000) databases were used for functional annotation of predicted gene models. tRNAscan‐SE v1.3.1 (Chan and Lowe, 2019) was used to annotate tRNAs, and BLASTN v2.2.31 was used to identify rRNAs. Rfam/Infernal v1.1 (Nawrocki and Eddy, 2013) was used to predict microRNAs (miRNAs) and small nuclear RNAs (snRNAs) in the genome. Additionally, the functional annotation of genes was performed using eggNOG‐mapper v‐2.1.12‐1/eggNOG DB v5.0.2 (Cantalapiedra *et al*., 2021; Huerta‐Cepas *et al*., 2018). We then used a custom script for gene GO and KEGG annotations.

### Phylogenomic analysis and detection of gene family expansion and contraction

We used protein‐coding genes to analyse the phylogenetic relationships between *C. tracyanum* and 18 species, including *Acorus gramineus*, *Amborella trichopoda*, *Ananas comosus*, *Apostasia shenzhenica*, *Arabidopsis thaliana*, *Asparagus officinalis*, *Dendrobium nobile*, *Elaeis guineensis*, *Gastrodia elata*, *Liriodendron tulipifera*, *Nymphaea colorata*, *Oryza sativa*, *Phalaenopsis equestris*, *Rosa chinensis*, *Solanum lycopersicum*, *Vitis vinifera*, *Zostera marin*a, *C. mannii* and *C. tracyanum* (Table S7). OrthoFinder v2.5.5 (Emms and Kelly, 2019) was used to perform ortholog inference analysis. As a result, a total of 26 697 gene clusters were identified, including 351 one‐to‐one single‐copy families.

We aligned and trimmed each single‐copy orthogroup. Specifically, the sequences in each orthogroup were aligned by MAFFT v7.505 (Katoh and Standley, 2013) and trimmed by Gblocks v0.91b (Talavera and Castresana, 2007). Subsequently, IQ‐TREE 2 v2.2.2.7 was used with all 351 aligned loci to estimate a maximum likelihood concatenated tree with 1000 bootstrap replicates (Minh *et al*., 2020). Then, the MCMCtree program within PAML v4.10.7 was used to determine the divergence times within the generated phylogenetic tree (Yang, 2007). Calibration points, obtained from publications and the TimeTree website (http://www.timetree.org), were utilized as normal to constrain the age of the nodes between *A. thaliana* and *R. chinensis* (102.0–112.5 Mya), *A. comosus* and *O. sativa* (94.1–117.0 Mya), *C. mannii* and *C. tracyanum* (9.3–45.0 Mya), *C. tracyanum* and *D*. *nobile* (12.3–51.0 Mya), *C. tracyanum* and *A. officinalis* (92.5–118.5 Mya), *C. tracyanum* and *A. shenzhenica* (72.2–114.1 Mya), *C. tracyanum* and *A. trichopoda* (179.9–205.0 Mya), *C. tracyanum* and *N. colorata* (168.4–191.6 Mya).

The expansions and contractions of gene families were identified through a comparison of the differences in cluster size between the ancestor and each species employing CAFÉ v5.1 (Mendes *et al*., 2020). The phylogenetic relationships, divergence times and expansions and contractions of gene families were visualized and edited with the assistance of iTOL v5 (Letunic and Bork, 2021).

### Collinearity analysis and whole‐genome duplication

Paralogs and orthologs were detected using the Best Reciprocal Hit (BRH) method. Firstly, the proteomes of five orchids (*A. shenzhenica*, *P. equestris*, *C. tracyanum*, *C. mannii* and *D. nobile*) were aligned by BLASTP v2.15.0 (Camacho *et al*., 2009) with an E‐value of a maximal 1e^−10^ to search all potential homologous gene pairs of protein sequences. The resulting BLASTP outputs and GFF annotation files of the genomes were processed using *MCScanX* v2 (Wang *et al*., 2012) with default parameters to assess the duplications within a species genome and syntenic regions between two different species. All orthologs defined by the BRH method had their corresponding protein‐coding DNA sequences (CDS) aligned using MAFFT v7.526 with default parameters (Katoh and Standley, 2013). The number of synonymous substitutions per synonymous site (*K*s) for each BRH was calculated by KaKs_Calculator v3.0 using the approximate Nei‐Gojobori method (Zhang, 2022). The *K*s distribution was plotted using the ggplot package in R v4.3.2 with the density function.

Synteny plots were drawn based on the results of the previous genome synteny blocks between *C. tracyanum* and *C. mannii*, *C. tracyanum* and *D. nobile*. Meanwhile, MCscan (Python version) was used to complement the assessment (https://github.com/tanghaibao/jcvi/wiki/MCscan‐(Python‐version)) (Tang *et al*., 2008). Specifically, the LAST output was filtered to remove tandem duplications and weak hits. Single linkage clustering was performed on the LAST output to cluster anchors into synteny blocks (Kielbasa *et al*., 2011).

The BLASTP results of all protein sequences in the *C. tracyanum* genome, along with the GFF annotation file, were input into *MCScanX*. The duplicate_gene_classifier module was then employed to identify and classify the gene duplication modes (Wang *et al*., 2012).

### Identification of TPS gene family

We identified TPS gene families from the genomes of 10 species (*A. shenzhenica*, *A. thaliana*, *C. mannii*, *C. tracyanum*, *D. nobile*, *O. sativa*, *P. equestris*, *Populus trichocarpa*, *S. moellendorffii* and *S. lycopersicum*). The hidden Markov models of PF01397 (N‐terminal) and PF03936 (C‐terminal) (Starks *et al*., 1997) were downloaded from the Pfam database (http://pfam.xfam.org/), and HMMER v3.1b2 software was used to search the TPS gene family sequences. BLAST was utilized to search for genes homologous to *TPS* genes in *A. thaliana*. These results were combined to identify *TPS* genes. For *Abies grandis*, due to the lack of genome information, we referred to the results reported in other studies to obtain *TPS* gene sequences (Huang *et al*., 2021; Yu *et al*., 2020). The expression heatmaps and distribution of *TPS* genes on chromosomes were visualized by TBtools (Chen *et al*., 2023).

### Volatolomics analysis of floral volatile compounds

Different parts of *C. tracyanum* flowers at the full‐blooming stage and the petals of *C. lowianum* at the bud and full‐blooming stages were frozen, and then ground into powder. A total of 50 ± 1 mg of each sample was placed into a 20‐mL headspace bottle, and 10 μL of 2‐Octanol (10 mg/L) was added as an internal standard. In the SPME cycle of the PAL rail system, samples were incubated at 60 °C for 30 min, following a 15‐min pre‐heat. the desorption time was 4 min. GC–MS analysis was performed using an Agilent 7890 gas chromatograph system coupled with a 5977B mass spectrometer (Agilent technologies, CA, USA). The system utilized a DB‐Wax and was injected in splitless mode. Helium was used as the carrier gas. The gas flow rate through the column was 1 mL/min. The initial temperature was kept at 40 °C for 4 min, then raised to 245 °C at a rate of 5 °C/min, and kept for 5 min. Chroma TOF 4.3X software of LECO Corporation (MI, USA) and the NIST database (National Institute of Standards and Technology, MD, USA) were used for raw peak extraction, data baseline filtration and calibration, peak alignment, deconvolution analysis, peak identification, integration and spectrum matching of the peak area. Principal component analysis (PCA) of the identified metabolites was performed using the R package (www.r‐project.org). Based on the variable importance in projection (VIP) scores obtained from the OPLS‐DA model, metabolites with VIP ≥1.0 and *P*‐value ≤0.05 were defined as significantly changed metabolites (SCMs).

### Qualitative and quantitative analysis of volatile terpenoids in fresh flowers of *C. tracyanum*

Floral volatiles were analysed using HS‐SPME‐GC‐MS in HP6890GC/5973MS system (Agilent Technologies, CA, USA). Briefly, fresh flowers of *C. tracyanum* at six developmental stages were excised, enclosed in clean glass vials, and heated in a 45 °C water bath for 20 min. Then, a 50/30 μm DVB/CAR/PDMS extraction fibre, fixed on an SPME holder (Sigma‐Aldrich, MA, USA) was inserted into the headspace of the glass vial and extracted for 40 min before injection. The GC was equipped with an HP‐5MS column (30 m × 0.25 mm × 0.25 μm). Temperature was held at 40 °C for 2 min, raised to 160 °C at 2.5 °C/min and then raised to 280 °C at 15 °C/min. The injector and detector temperatures were maintained at 250 °C. The carrier gas helium flow rate was 1.0 mL/min. Identification of HS‐SPME‐GC–MS was performed by comparison with n‐alkane standards and the NIST 14 (National Institute of Standards and Technology, MD, USA) mass spectral library. Retention indices (RI) of the compounds were determined by using the Kovat index. RI = 100_*n*_ + 100 [T_R(x)_ − T_R(*n*)_]/[T_R(*n*+1)_ − T_R(*n*)_], where T_R(x)_, T_R(*n*)_ and T_R(*n*+1)_ represent the retention times of the compound and the normal alkanes with carbon numbers *n* and (*n* + 1), respectively. GC–MS data of volatile compounds values were shown as means ± SD of triplicates. The standard was diluted with dichloromethane solution in five sequential gradients from the stock solution, and the standard curve was calculated. The characteristic ion peak area response value of the target volatile compound was substituted into the standard curve to calculate the content (Table S22). The odour activity value (OAV) was calculated by the following formula: OAV = C/OT, where C is the concentration of the volatile compound and OT is its odour threshold. The odour threshold values have been cited from *Odour thresholds: compilations of odour threshold values in air, water and other media* (van Gemert, 2011). Compounds with OAV ≥1 were considered potential contributors to the floral scent profile. In general, higher OAV values indicate compounds that contribute more significantly to the volatile profile.

### Transcriptome construction, assembly and annotation

Total RNA was extracted from petals of *C. tracyanum* and *C. lowianum* at different developmental stages, as well as from different parts of full‐blooming flowers of *C. tracyanum* (three biological replicates). Raw data were obtained using the Illumina Novaseq 6000 platform. Raw data in fastq format were first processed through in‐house Perl scripts, and clean data were obtained by removing reads containing adapter, reads containing N bases and low‐quality reads from the raw data. Then paired‐end clean reads were aligned to the reference genome using HISAT2 v2.0.5 (Kim *et al*., 2019). featureCounts v1.5.0‐p3 (Liao *et al*., 2013) was used to count the read numbers mapped to each gene. FPKM values of each gene were calculated based on the length of the gene and the read count mapped to the gene. Differentially expressed genes (DEGs) were screened by |log_2_(foldchange)| ≥ 1 and padj ≤0.05 by applying *DESeq2* R package 1.20.0 (Love *et al*., 2014).

### Enrichment analysis

To further understand the functions of expanded and contracted genes in the *C. tracyanum* genome and differentially expressed genes in the transcriptome, we first collated GO and KEGG annotations of these genes. Then, clusterProfiler 4.0 (Wu *et al*., 2021) was used to perform enrichment analysis and visualization.

### Identification of structural genes and TFs related to terpenoid biosynthesis

We identified genes encoding enzymes related to terpenoid biosynthesis according to the KEGG annotation (https://www.genome.jp/kegg/annotation/). The related genes in the transcriptomes were identified by using a local TBLASTN algorithm with an *E*‐value cut‐off of 1e^−5^ and confirmed using BLAST on the National Center for Biotechnology Information website (https://www.ncbi.nlm.nih.gov). To isolate TFs and regulators, all proteins obtained from transcriptomes were annotated and classified by using iTAK (Zheng *et al*., 2016). Among these, 642 genes annotated as bHLH, bZIP, EIL, NAC, MYB, WRKY and AP2/ERF were isolated. Heatmaps of genes related to terpenoid biosynthesis pathways were generated by TBtools (Chen *et al*., 2023).

### Weighted gene co‐expression network analysis (WGCNA)

WGCNA was performed using the WGCNA R package (Langfelder and Horvath, 2008). We first screened genes from the transcriptome of petals of *C. tracyanum* at different developmental stages, selecting the top 75% with a median absolute deviation ≥0.01. After filtering, the abundance of 25 364 genes and 12 terpenoid metabolites was used to build a co‐expression network by calculating correlation coefficients. The soft threshold power of the correlation network was set to 18, the minimum module size was set to 30, and the cutHeight for merging modules was set to 0.5. The eigengene value was calculated for each module and used to test the association with each metabolite. The co‐expression network was visualized with Cytoscape v3.8.2 (Shannon *et al*., 2003).

### Quantitative real‐time PCR analysis

All qRT‐PCR primers were designed using the Primer Premier 6.0 program (Primer Biosoft Inc., QC, Canada) and are listed in Table S21. Quantitative real‐time PCR was carried out using the Bio‐Rad real‐time PCR System (Bio‐Rad, CA, USA). We configured the reaction mixtures according to the manufacturer's instructions of iTaq Universal SYBR® Green Supermix (Bio‐Rad, CA, USA). Relative gene expression was calculated using the 2^−ΔΔCт^ method (Livak and Schmittgen, 2001). Three biological replicates were used for each analysis.

### Subcellular localization of CtTPS proteins

The intact ORF sequences of *CtTPS* genes were integrated into a pCAMBIA Super 1300‐eGFP vector by replacing the termination codons with sequences encoding enhanced green fluorescent protein (eGFP), under the control of a mannopine synthetase (mas) promoter (Table S21). The resulting vector construct was transformed into *Agrobacterium tumefaciens* (strain GV3101) and used to infiltrate 4‐week‐old *N. benthamiana* leaves. Three days post‐infiltration, protoplasts were isolated from the tobacco leaves. Protoplasts expressing eGFP fusions of CtTPS proteins were visualized by confocal laser scanning microscopy LSM 900 (Carl Zeiss, Jena, Germany).

### *In vitro* characterization of recombinant CtTPS proteins

DNA fragments of *CtTPS* genes were inserted into a pET‐32a vector. The recombinant plasmids were then transformed into *E. coli* strain BL21 (DE3). Recombinant proteins were induced by the application of 0.2 mm isopropyl *β*‐D‐thiogalactoside (IPTG) at 16 °C overnight. Induced cells were harvested by centrifugation, resuspended in Tris‐buffered saline and disrupted by sonication on ice. After centrifugation, the supernatants were purified using Ni‐NTA Agarose (Qiagen, Venlo, Netherlands). Purified proteins were collected and concentrated before enzyme assays. The purity of the isolated proteins was verified by densitometry of SDS‐PAGE gels after Coomassie Brilliant Blue staining. Protein concentrations were estimated using the Detergent Compatible Bradford Protein Assay Kit (Beyotime, Shanghai, China).

Assays for recombinant CtTPS protein activity were carried out in a 500 μL assay buffer (50 mm HEPES, pH 7.2, 10% [v/v] glycerol, 10 mm MgCl_2_, 1.25 mm MnCl_2_, 5 mm DTT) containing 10 μg purified recombinant CtTPS proteins and 20 μg GPP/FPP. The mixture was incubated at 30 °C for 2 h and then mixed vigorously at 60 °C for 5 min to obtain enzymatic products. The catalytic products were collected using the DVB/CAR/PDMS headspace sampler and analysed by GC–MS. Extracts from *E. coli* transformed with the pET‐32a empty vector served as controls and standard samples were analysed under the same conditions (Table S19). Unfortunately, CtTPS1 failed to be induced as a soluble protein in the supernatant, and therefore, no enzyme activity assay was performed.

### Dual‐luciferase assays

The full‐length CDS of *CtTF* genes was cloned into the pM999‐eGFP vector under the control of the CaMV 35S promoter as effectors. The promoter fragments of *CtTPS* genes were ligated into the binary vector pGreenII‐0800‐LUC as the double‐reporter vector (Hellens *et al*., 2005). The pM999‐eGFP vector without *CtTF* genes was used as a negative control. Primers used in this assay are listed in Table S21. The constructed effector and reporter vectors were co‐transformed into tobacco (*N. benthamiana*) leaf protoplasts using the polyethylene glycol 4000 (PEG 4000) method, as previously described (Abel and Theologis, 1994). The transformed protoplasts were incubated at 23 °C for 16 h in darkness, and dual‐luciferase assays were performed using the Dual‐Luciferase® Reporter Assay System (Vazyme, Nanjing, China). Luciferase activity was measured using the Infinite® 200 PRO plate reader (TECAN Group, Switzerland). Finally, the LUC:REN ratio was calculated and normalized to the control vector as the final value. The pM999‐eGFP empty vector was used as a negative control. At least three replicates were used for each dual‐luciferase assay.

### Yeast one‐hybrid assays

Promoter fragments within 2 kb of *CtTPS* genes were amplified via PCR using corresponding primers (Table S21). The amplified products were verified by sanger sequencing and cloned into the pAbAi vector as the baits, and the full‐length CDS of *CtTF* genes were subcloned from the pM999‐eGFP vector into the pGADT7 vector to construct the prey. The linearized recombinant pAbAi vector was first transformed into the Y1H Gold yeast strain via the homologous recombination method and selected on SD/‐Ura medium. Subsequently, the recombinant pGADT7 vector was introduced into the yeast strain, and transformants were further selected for resistance concentrations using SD/‐Leu/‐Ura medium with proper concentrations of aureobasidin A. According to the growth ability of the yeast colonies, the protein‐DNA interaction was determined. The pGADT7 vector without the *CtTF* gene sequence was used as a negative control.

### Electrophoretic mobility shift assays

Electrophoretic mobility shift assays (EMSAs) were performed using an EMSA/Gel‐Shift kit (Beyotime) according to the manufacturer's instructions. The coding sequences of *CtAP2/ERF1*, *CtbZIP1*, *CtMYB2*, *CtMYB3* and *CtAP2/ERF4* were amplified and cloned into the pET‐32a vector for fusion with a His tag. The purification of recombinant proteins was performed as described in the section “*In vitro* characterization of recombinant CtTPS proteins”. Oligonucleotide probes were 5′ end‐labelled with biotin. For competition assays, unlabeled competitors were added to the reaction at 50‐ and 200‐fold (sequences are listed in Table S21). The DNA–protein complexes were separated by gel electrophoresis and transferred to a nylon membrane (GE Healthcare, Chicago, IL, USA). After UV crosslinking, the biotin signal was detected according to the manufacturer's instructions. Experiments were repeated at least twice with consistent results.

### Statistical analysis

One‐way ANOVA was used to determine the significance of gene expression levels at different developmental stages and in different parts of *C*. *tracyanum*. The threshold for statistical significance was set at *P* < 0.05. Statistical analysis of gene expression levels between *C. tracyanum* and *C. lowianum* was performed using Student's *t*‐test (*P < 0.05, **P < 0.01 and ***P < 0.001). For the dual‐luciferase assay, the significance of the LUC/REN ratio was calculated by Student's *t*‐test compared with the negative control (*P < 0.05, **P < 0.01 and ***P < 0.001). Data are presented as means (±SD) from three replicates. All statistical analyses were performed using R software.

## Author contributions

S.‐B.Z., A.Z. and J.‐B.Y. conceived the project and designed the research. M.T., N.L. and Z.‐S.H. performed the analyses of the genome and transcriptome sequence. M.T., X.‐M.D. and T.‐Y.G. collected the samples and performed the experiments. M.T., N.L. and Z.‐S.H. wrote the manuscript. S.‐B.Z., A.Z. and J.‐B.Y. revised the manuscript. All authors read and approved the final manuscript.

## Funding

This work was supported by the National Key Research and Development Program of China (2024YFF1306703), the National Natural Science Foundation of China (32170393), the Strategic Priority Research Program of the Chinese Academy of Sciences (XDB31000000), the Key Research and Development Program of Yunnan Province (202403AC100032), the Yunnan Fundamental Research Project (202201AU070123 and 202301AT070306) and the High‐level Talent Support Plan of Yunnan Province (YNWR‐CYJS‐2020‐023).

## Competing interests

The authors declare that they have no competing interests.

## Supporting information


**Table S1** Summary of sequenced data from multiple platforms in this study.

**Table S2** Summary of *C. tracyanum* genome assemblies.

**Table S3** Repetitive elements identified in *C. tracyanum* genome.

**Table S4** Summary of annotated protein‐coding genes in *C. tracyanum*.

**Table S5** Functional annotation in *C. tracyanum* genome.

**Table S6** BUSCO estimates of gene annotation completeness.

**Table S7** Species information used in phylogenomic analysis.

**Table S8** GO enrichment analysis of the expanded genes in *C. tracyanum* genome.

**Table S9** *TPS* genes used for phylogenetic tree construction.

**Table S10** Duplication types of *TPS* genes in the genome of *C. tracyanum*.

**Table S11** Summary of the 230 detected metabolites in volatolomics.

**Table S12** Differential metabolites in *C. tracyanum* and *C. lowianum* petals at the full‐blooming stage.

**Table S13** Terpenoids detected in fresh flowers of *C. tracyanum* at six developmental stages.

**Table S14** Transcriptome reads mapping rate.

**Table S15** The genes annotated as putative enzymes involved in the terpenoid biosynthesis pathway.

**Table S16** Expression statistics of genes with an average FPKM >1 in different floral parts at the full‐blooming stage of *C. tracyanum*.

**Table S17** Information about candidate *CtTPS* genes, transcription factors and reference genes.

**Table S18** TPS proteins from other plant species used in phylogenetic analysis.

**Table S19** *In vitro* enzymatic reaction products and authentic standard information.

**Table S20** GO enrichment analysis of the contracted genes in *C. tracyanum* genome.

**Table S21** Primers and probes used in this study.

**Table S22** Information about standard curves.

**Figure S1** Hi‐C map of the *C. tracyanum* genome showing genome‐wide all‐by‐all interactions.
**Figure S2** Estimation of *C. tracyanum* genome size by flow cytometry and *k*‐mer analysis.
**Figure S3** The estimated time distribution of LTR‐RTs insertions.
**Figure S4** Gene pairs in the synteny region among *C. tracyanum*, *C. mannii* and *D. nobile*.
**Figure S5** Volatile compound profiles in *C. tracyanum* and *C. lowianum*.
**Figure S6** Gas chromatogram of floral volatiles from the fresh flowers of *C. tracyanum* and *C. lowianum*.
**Figure S7** Scanning Electron Microscope (SEM) images of different parts of *C. tracyanum* flowers.
**Figure S8** Enrichment analysis of differentially expressed genes in the petals of *C. tracyanum* at Bud and D+1 stages.
**Figure S9** Schematic representation of the distribution of *TPS* genes on *C. tracyanum* chromosomes and together with the locations of key genes involved in terpenoid biosynthesis.
**Figure S10** Expression levels of genes encoding key enzymes in the terpenoid backbone biosynthesis pathway in the petals of *C. tracyanum* and *C. lowianum* at different developmental stages analyzed by qRT‐PCR.
**Figure S11** Phylogenetic analysis of HMGR proteins from *C. tracyanum* and other plants.
**Figure S12** Phylogenetic analysis of DXS proteins from *C. tracyanum* and other plants.
**Figure S13** Expression profiles of putative genes encoding enzymes for terpenoid biosynthesis in different parts of *C. tracyanum* flowers at full‐blooming stage.
**Figure S14** Heatmap of the expression patterns of *TPS* genes from different parts of *C. tracyanum* flowers at the full‐blooming stage.
**Figure S15** Hierarchical cluster tree displaying 25 modules of co‐expressed genes.
**Figure S16** Expression levels of eight *CtTPS* genes in different parts of *C. tracyanum* flowers (at the D+15 stage) by qRT‐PCR analysis.
**Figure S17** Expression levels of candidate TFs in different parts of *C. tracyanum* flowers (at the D+15 stage) by qRT‐PCR analysis.
**Figure S18** SDS‐PAGE analysis of recombinant terpene synthase proteins.
**Figure S19** Subcellular localization of free eGFP and six CtTF‐eGFP fusions in tobacco leaf protoplasts.
**Figure S20** SDS‐PAGE analysis of recombinant transcription factor proteins.
**Figure S21** GO pathway enrichment distribution of the contracted genes in *C. tracyanum* genome.

## Data Availability

All raw sequencing reads have been deposited in the NCBI Sequence Read Archive (https://www.ncbi.nlm.nih.gov/sra) under project PRJNA1145103.
