# Supplementary material for: Integrative omics reveals mechanisms of biosynthesis and regulation of floral scent in Cymbidium tracyanum
Source: Plant Biotechnol J. 2025 Mar 17;23(6):2162–81. doi: 10.1111/pbi.70025 (PMC12120893; doi:10.1111/pbi.70025)
Supplement: Supplementary file 23 — Figure S1 Hi‐C map of the C. tracyanum genome showing genome‐wide all‐by‐all interactions. Figure S2 Estimation of C. tracyanum genome size by flow cytometry and k‐mer analysis. Figure S3 The estimated time distribution of LTR‐RTs insertions. Figure S4 Gene pairs in the synteny region among C. tracyanum, C. mannii and D. nobile. Figure S5 Volatile compound profiles in C. tracyanum and C. lowianum. Figure S6 Gas chromatogram of floral volatiles from the fresh flowers of C. tracyanum and C. lowianum. Figure S7 Scanning Electron Microscope (SEM) images of different parts of C. tracyanum flowers. Figure S8 Enrichment analysis of differentially expressed genes in the petals of C. tracyanum at Bud and D+1 stages. Figure S9 Schematic representation of the distribution of TPS genes on C. tracyanum chromosomes and together with the locations of key genes involved in terpenoid biosynthesis. Figure S10 Expression levels of genes encoding key enzymes in the terpenoid backbone biosynthesis pathway in the petals of C. tracyanum and C. lowianum at different developmental stages analyzed by qRT‐PCR. Figure S11 Phylogenetic analysis of HMGR proteins from C. tracyanum and other plants. Figure S12 Phylogenetic analysis of DXS proteins from C. tracyanum and other plants. Figure S13 Expression profiles of putative genes encoding enzymes for terpenoid biosynthesis in different parts of C. tracyanum flowers at full‐blooming stage. Figure S14 Heatmap of the expression patterns of TPS genes from different parts of C. tracyanum flowers at the full‐blooming stage. Figure S15 Hierarchical cluster tree displaying 25 modules of co‐expressed genes. Figure S16 Expression levels of eight CtTPS genes in different parts of C. tracyanum flowers (at the D+15 stage) by qRT‐PCR analysis. Figure S17 Expression levels of candidate TFs in different parts of C. tracyanum flowers (at the D+15 stage) by qRT‐PCR analysis. Figure S18 SDS‐PAGE analysis of recombinant terpene synthase proteins. Figure S19 Subcellu [file PBI-23-2162-s002.docx]

**Supplementary Materials for**

**Integrative omics reveals mechanisms of biosynthesis and regulation of floral scent in *Cymbidium tracyanum***

Mengling Tu ^1, 4 †^, Ningyawen Liu ^2, 4 †^, Zheng-Shan He ^3 †^, Xiu-Mei Dong ^1^, Tian-Yang Gao ^1, 4^, Andan Zhu ^3^ *, Jun-Bo Yang ^3^ * and Shi-Bao Zhang ^1^ *

^1^ Key Laboratory for Economic Plants and Biotechnology, Kunming Institute of Botany, Chinese Academy of Sciences, Kunming, Yunnan 650201, China

^2^ National Key Laboratory of Genetic Evolution & Animal Models, Kunming Natural History Museum of Zoology, Kunming Institute of Zoology, Chinese Academy of Sciences, Kunming, Yunnan 650201, China

^3^ Germplasm Bank of Wild Species, Kunming Institute of Botany, Chinese Academy of Sciences, Kunming, Yunnan 650201, China

^4^ University of Chinese Academy of Sciences, Beijing 100049, China

^†^ These authors contributed equally to this work

* Correspondence: Andan Zhu ([zhuandan@mail.kib.ac.cn](mailto:zhuandan@mail.kib.ac.cn)), Jun-Bo Yang ([jbyang@mail.kib.ac.cn](mailto:jbyang@mail.kib.ac.cn)), Shi-Bao Zhang ([sbzhang@mail.kib.ac.cn](mailto:sbzhang@mail.kib.ac.cn))

**Supplementary Methods**

**Biochemical reagents**

All gene and fragment amplifications were carried out on a Bio-Rad T100 thermal cycler using Phanta^®^ Max Super-Fidelity DNA Polymerase (Vazyme, Nanjing, China). PCR products were purified using the FastPure^®^ Gel DNA Extraction Mini Kit (Vazyme, Nanjing, China). Plasmid purifications were performed using the E.Z.N.A^®^ Endo-Free Plasmid Mini Kit Ⅱ (Omega Bio-tek, Georgia, USA) according to the manufacturer’s instructions. Sabinene (B29124-100mg), Myrcene (B21632-0.5mL), α-Terpineol (B21963-20mg), α-Farnesene (T25236-5g) and Nerolidol (B20176-0.2ml) were purchased from Shanghai yuanye Bio-Technology Co., Ltd (CHN); α-Phellandrene (Y55060-25g), (-)-Verbenone (DZP-B29019-100mg) and (*E*)-β-Caryophyllene (DZP-RS07271081) were purchased from Guangzhou keli technology development Co., Ltd (CHN); Terpinolene (T819532-100ml), Linalool (L812404-25ml), (-)-Terpinen-4-ol (T863238-25ml), α-Terpinene (A833311-25ml), α-Pinene (P823474-5ml), D-Limonene (R871932-5ml) and Neo-allo-ocimene (N775958-100g), were purchased from MACKLIN (CHN); Valencene (75056-10G-F), GPP (Product No. G6772), FPP (Product No. F6892), and IPTG (Product No. I6758) were purchased from Sigma-Aldrich (USA). Aureobasidin A (Product Code: 630466) was obtained from Takara Bio.

**Plant Materials**

All plants used in this study were cultivated in the greenhouse at the Kunming Institute of Botany, Kunming, China (25°14′ N, 102°74′ E). Wild-type tobacco plants (*Nicotiana benthamiana*) for dual-luciferase reporter assays and subcellular localization in protoplasts were grown in a greenhouse at 22°C with a photoperiod of 16 h/8 h (light/dark). Leaves from 3- to 4-week-old plants were used for protoplast isolation.

**DNA or RNA extraction and cDNA synthesis**

In order to obtain the genomic sequences of the *CtTPS* genes, DNA was extracted from the flowers of *C. tracyanum* using the EasyPure^®^ Plant Genomic DNA Kit (TransGen, Beijing, China) according to the manufacturer’s instructions. RNA was extracted from samples by using the Biospin^®^ RNA kit (DNase I) (BioFlux, Hangzhou, China) in accordance with the manufacturer’s protocol. The concentration and quality of RNA were measured with the NanoDrop™ One Spectrophotometer (NanoDrop Technologies, ThermoScientific, MA, USA). Approximately 1 μg of total RNA from each sample was reverse transcribed into first strand cDNA with a final volume of 20 μL using the ThermoScientific™ RevertAid H Minus Reverse Transcriptase Kit (ThermoScientific, MA, USA) according to the manufacturer’s instructions.

**Gene cloning and sequence analysis**

Full-length gene sequences of *CtTPS* and *CtTF* genes were amplified by PCR from cDNA of *C. tracyanum*. To amplify the promoter region of *CtTPS* genes, combinations of primers were designed and used in PCR reactions with genomic DNA as templates. All primers used in this study were listed in Table S21.

To elucidate the phylogeny of *CtTPS* genes, the full-length amino acid sequences of CtTPS proteins and their homologs in other plants that have been verified functions were aligned using MAFFT v7.505 with parameter —maxiterate 1000 —localpair, and the alignment was trimmed using trimAl v1.4 with default parameters. The gene tree was inferred using IQ-TREE 2 with maximum likelihood and visualized by iTOL v5.

**Scanning electron microscopy (SEM)**

Different areas of the perianth of fresh flowers were cut into 1 mm^3^ cubes and immediately fixed in glutaraldehyde. Water was gradually removed from the samples using ethanol solutions of different concentrations. After critical-point drying and sputter coating with gold, the samples were studied using the Zeiss Sigma 300 SEM (Carl Zeiss, Jena, Germany).

**Supplementary Tables**

Table S1 Summary of sequenced data from multiple platforms in this study.

Table S2 Summary of *C. tracyanum* genome assemblies.

Table S3 Repetitive elements identified in *C. tracyanum* genome.

Table S4 Summary of annotated protein-coding genes in *C. tracyanum*.

Table S5 Functional annotation in *C. tracyanum* genome.

Table S6 BUSCO estimates of gene annotation completeness.

Table S7 Species information used in phylogenomic analysis.

Table S8 GO enrichment analysis of the expanded genes in *C. tracyanum* genome.

Table S9 *TPS* genes used for phylogenetic tree construction.

Table S10 Duplication types of *TPS* genes in the genome of *C. tracyanum*.

Table S11 Summary of the 230 detected metabolites in volatolomics.

Table S12 Differential metabolites in *C. tracyanum* and *C. lowianum* petals at full-blooming stage.

Table S13 Terpenoids detected in fresh flowers of *C. tracyanum* at six developmental stages.

Table S14 Transcriptome reads mapping rate.

Table S15 The genes annotated as putative enzymes involved in the terpenoid biosynthesis pathway.

Table S16 Expression statistics of genes with an average FPKM > 1 in different floral parts at the full-blooming stage of *C. tracyanum*.

Table S17 Information about candidate *CtTPS* genes, transcription factors and reference genes.

Table S18 TPS proteins from other plant species used in phylogenetic analysis.

Table S19 *In vitro* enzymatic reaction products and authentic standard information.

Table S20 GO enrichment analysis of the contracted genes in *C. tracyanum* genome

Table S21 Primers and probes used in this study.

Table S22 Information about standard curves.

**Supplementary Figures**

**
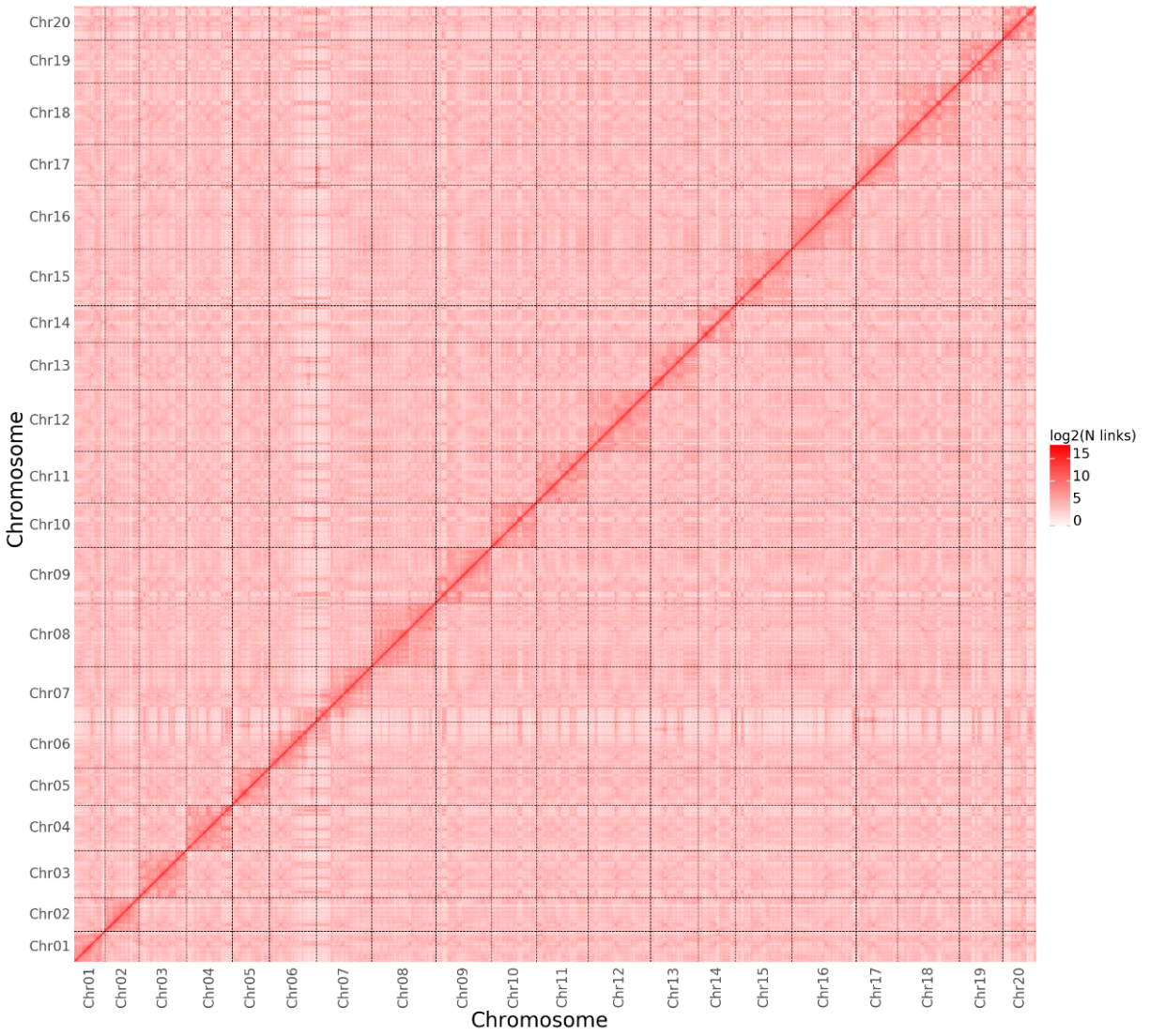
**

**Figure S1** **Hi-C map of the *C. tracyanum* genome showing genome-wide all-by-all interactions.** The interaction heatmap represents the normalized count of Hi-C links in 1Mb bins on a logarithmic scale.

**
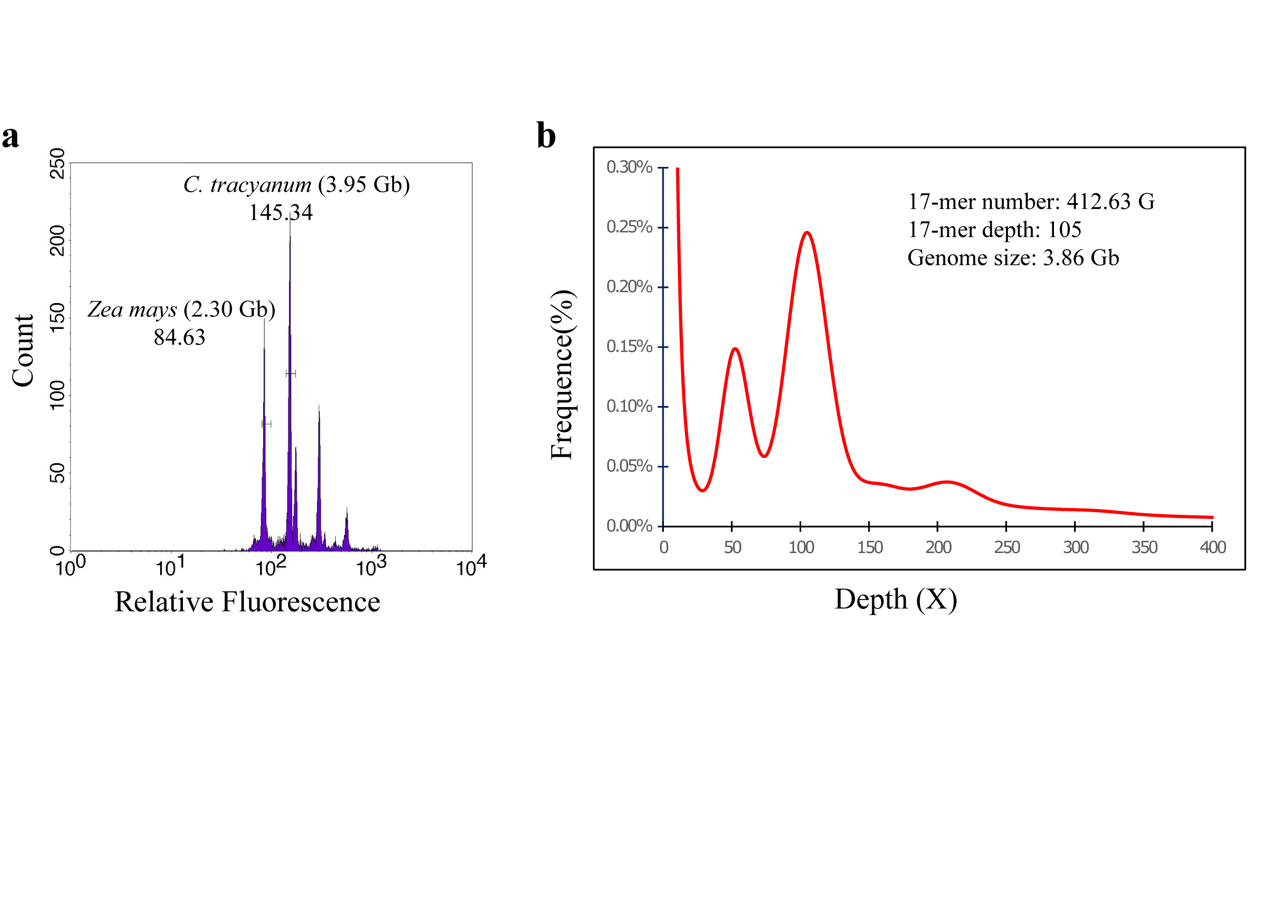
**

**Figure S2 Estimation of *C. tracyanum* genome size by flow cytometry (a) and *k*-mer analysis (b).** The reference species used was *Zea mays*, with a genome size of 2.30 Gb. The genome size of *C. tracyanum* is estimated to be approximately 3.95 Gb (a). The distribution curve of 17-mer. The x-axis represents the 17-mer depth, and the y-axis represents the proportion of *k*-mer frequency at that depth (b).


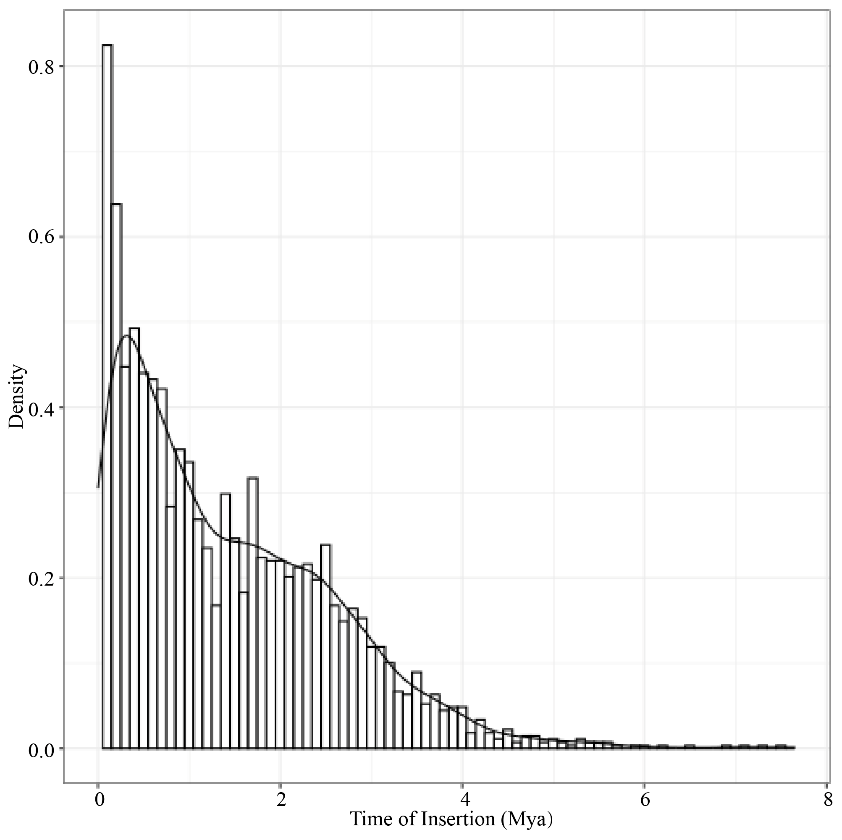


**Figure S3 The estimated time distribution of LTR-RTs insertions.** The x-axis represents the time of the insertion event, and the y-axis represents the frequency of insertion of LTR-RTs during the corresponding time period.


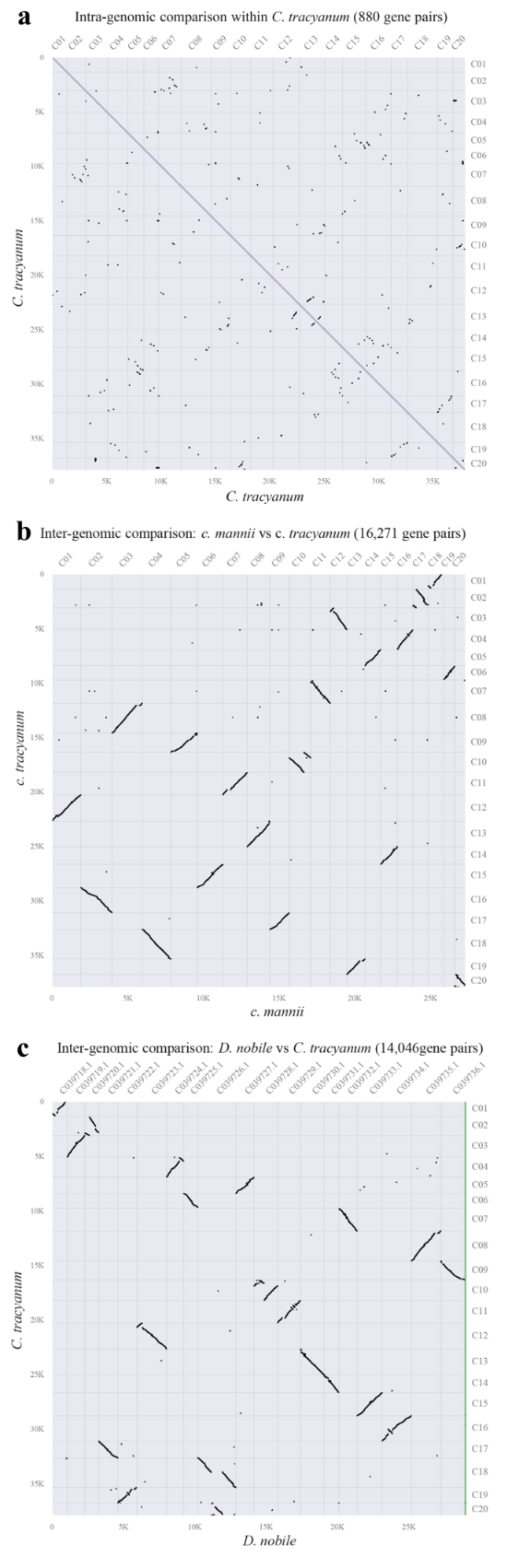


**Figure S4 Gene pairs in the synteny region among *C. tracyanum*, *C. mannii* and *D. nobile*.** (a) Analysis of intra-genomic synteny blocks of *C. tracyanum*. (b) Synteny blocks between *C. tracyanum* and *C. mannii* accounted for 48% of the genome. (c) Synteny blocks between *C. tracyanum* and *D. nobile* accounted for 40% of the genome.

**
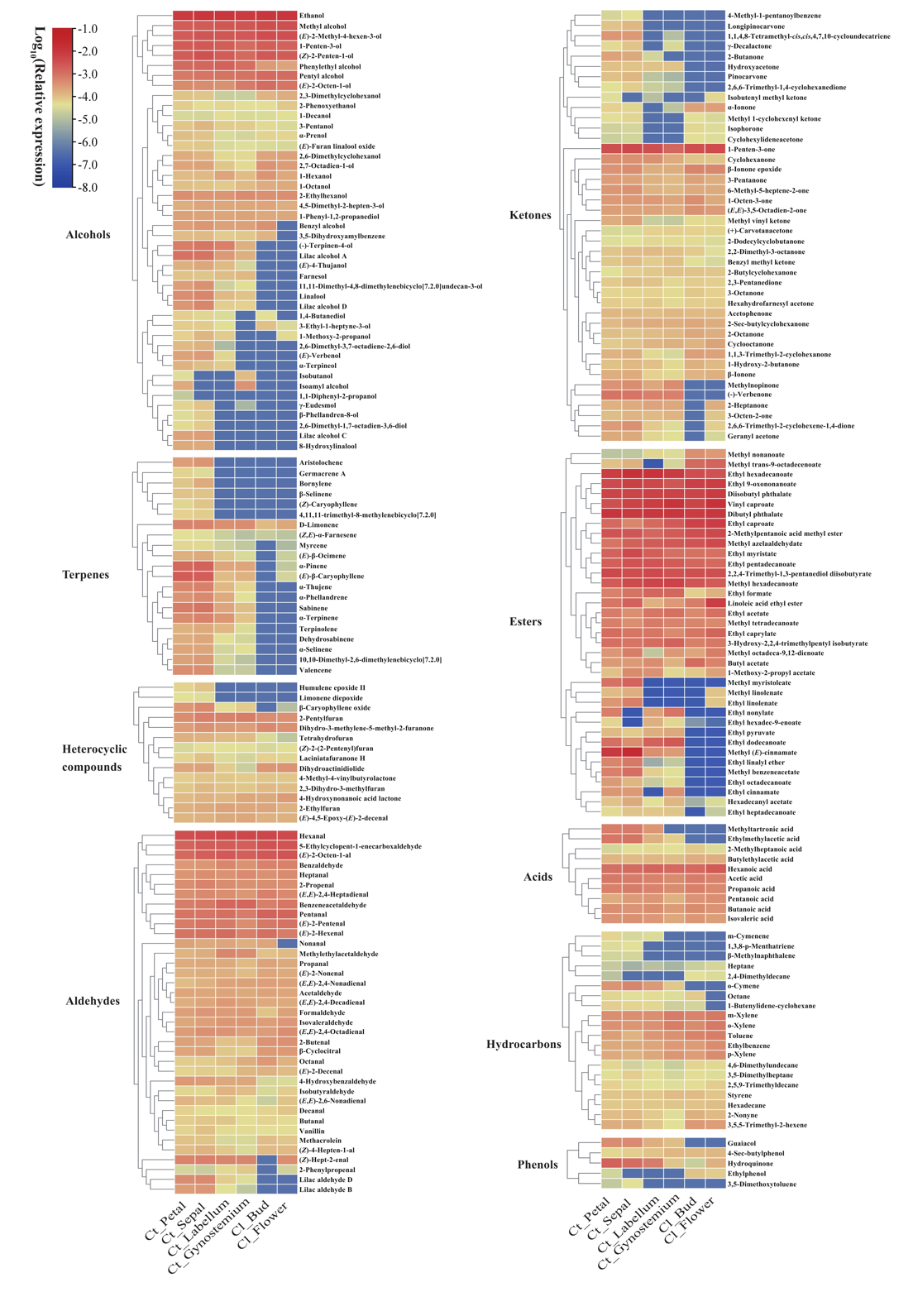
**

**Figure S5** **Volatile compound profiles in *C. tracyanum* and *C. lowianum*.** Composition and content of volatile compounds detected in volatolomics of different parts of *C. tracyanum* flowers at the full-blooming stage, and the petals of *C. lowianum* at the bud and full-blooming stages (see Table S11).

**
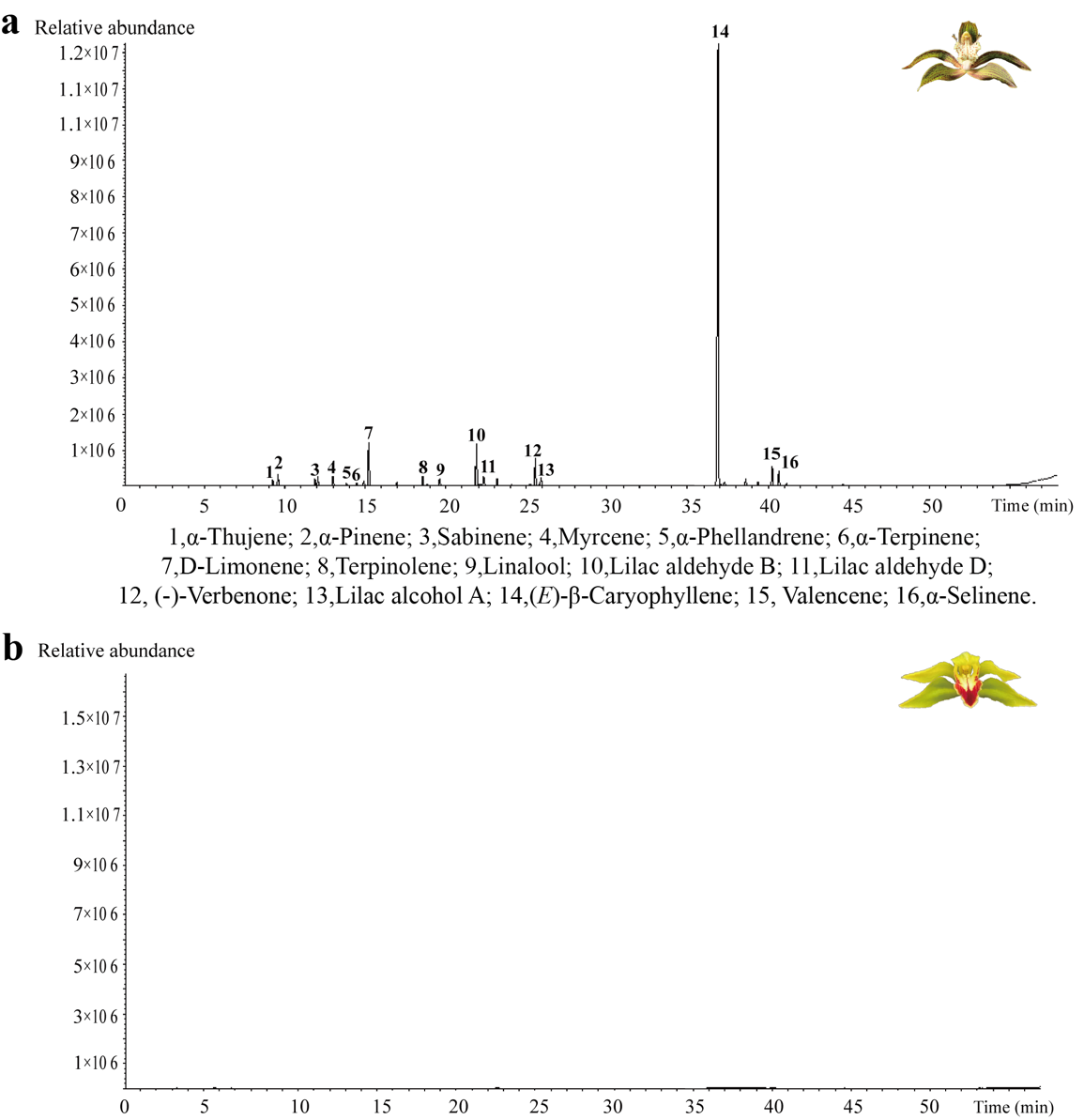
**

**Figure S6 Gas chromatogram of floral volatiles from the fresh flowers of *C. tracyanum* (a) and *C. lowianum* (b).** The major peaks are labeled with numbers on the chromatogram, and the corresponding compounds are listed below in order. Floral volatiles are measured three times independently with similar results.

­
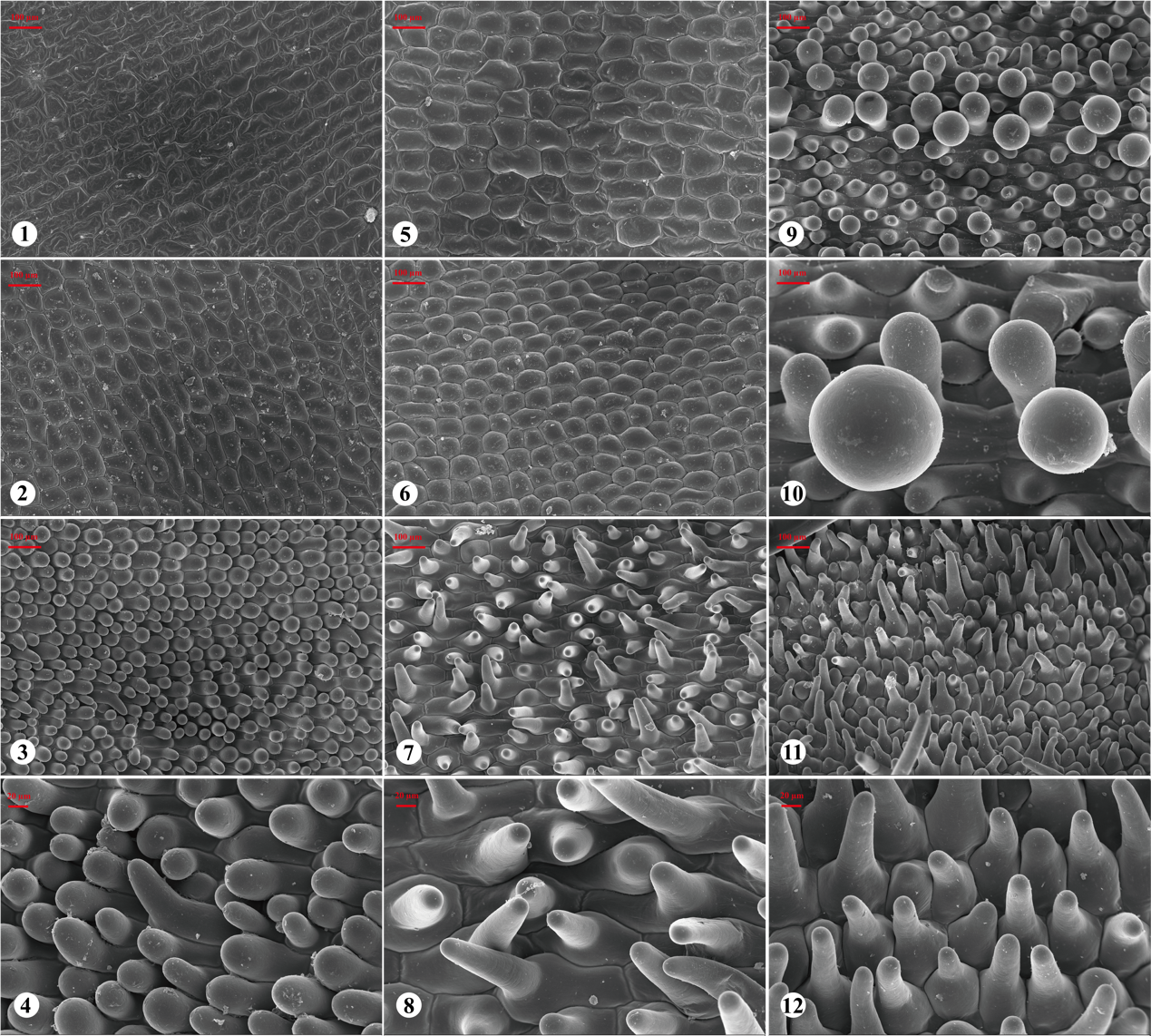


**Figure S7 Scanning Electron Microscope (SEM) images of different parts of *C. tracyanum* flowers.** 1-4, apical, medial, basal and partial enlargement of basal SEM images of the adaxial surface of the petal, respectively; 5-8, apical, medial, basal and partial enlargement of basal SEM images of the adaxial surface of the sepal, respectively; 9-10, adaxial surface and partial enlargement SEM images of the gynostemium; 11-12, adaxial surface and partial enlargement SEM images of the labellum; The cells at the base of the petals and sepals are considered to be immature and therefore exhibit a different morphology compared to the apical and middle epidermal cells.


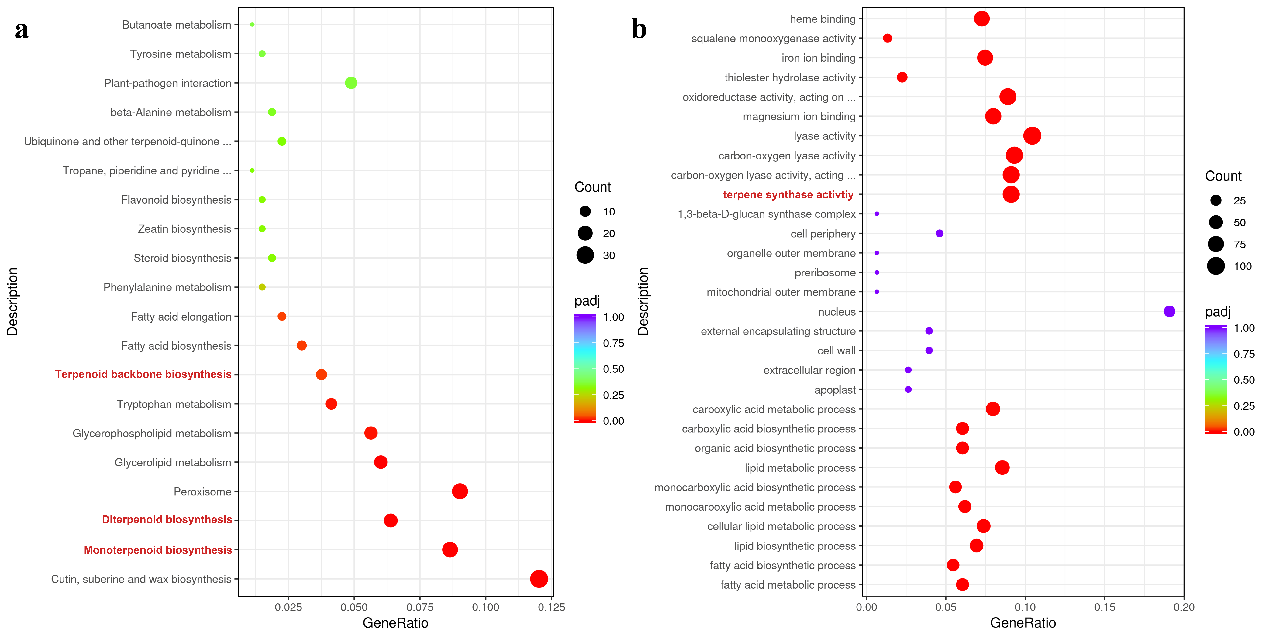


**Figure S8 Enrichment analysis of differentially expressed genes in the petals of *C. tracyanum* at Bud and D+1 stages.** (a) KEGG pathway enrichment distribution of differentially expressed genes; (b) GO functional enrichment of differentially expressed genes.


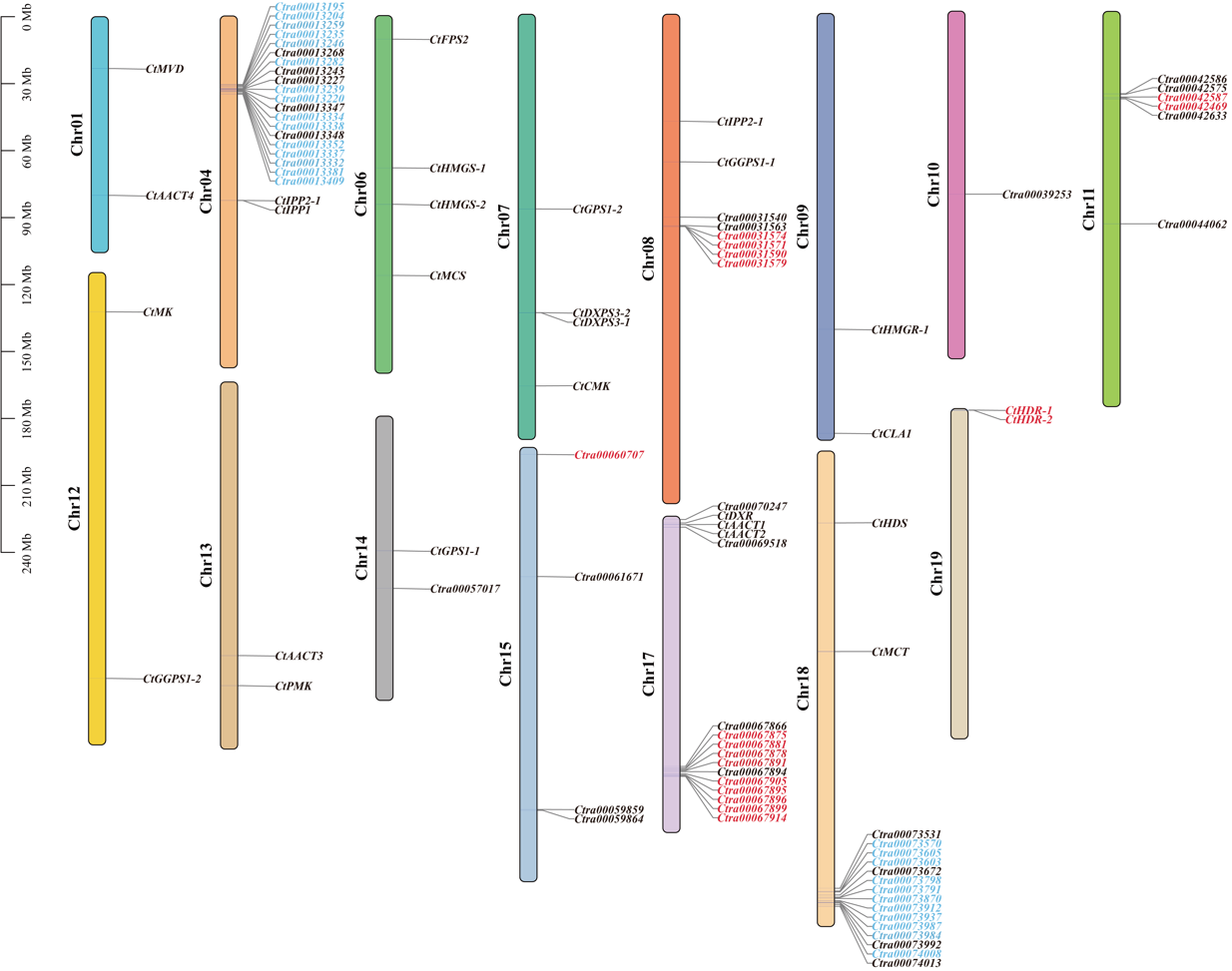


**Figure S9** **Schematic representation of the distribution of *TPS* genes on *C. tracyanum* chromosomes and together with the locations of key genes involved in terpenoid biosynthesis.** Blue and red labels indicate segmental and tandem duplications, respectively (see Table S10).


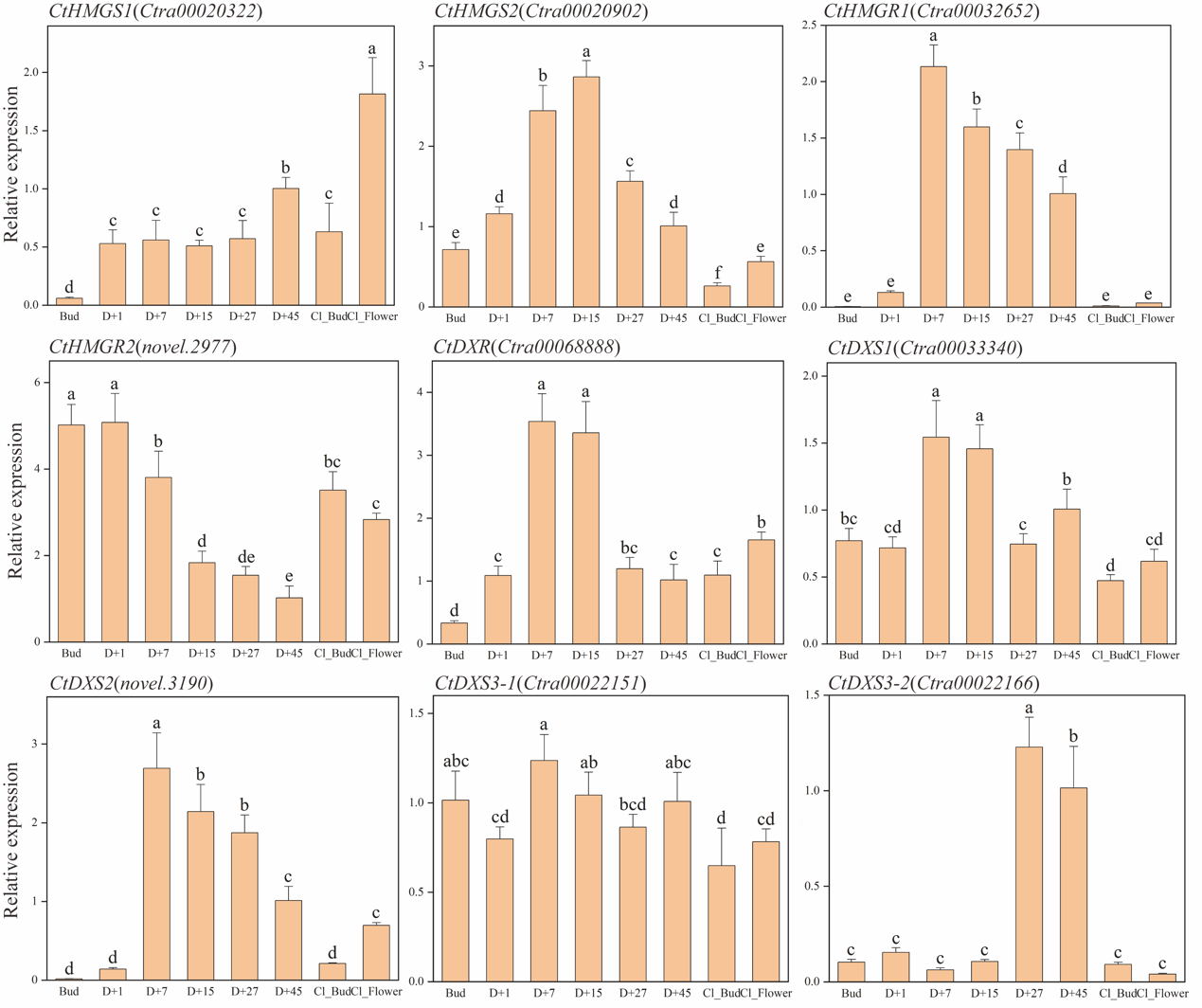


**Figure S10 Expression levels of genes encoding key enzymes of terpenoid backbone biosynthesis pathway in the petals of *C. tracyanum* and *C. lowianum* at different developmental stages analyzed by qRT-PCR.** *CtGAPDH* is quantified as internal controls. Different letters stand for significant differences in different stages calculated through one-way ANOVA (*P* < 0.05). Error bars stand for the SD of three replicates.


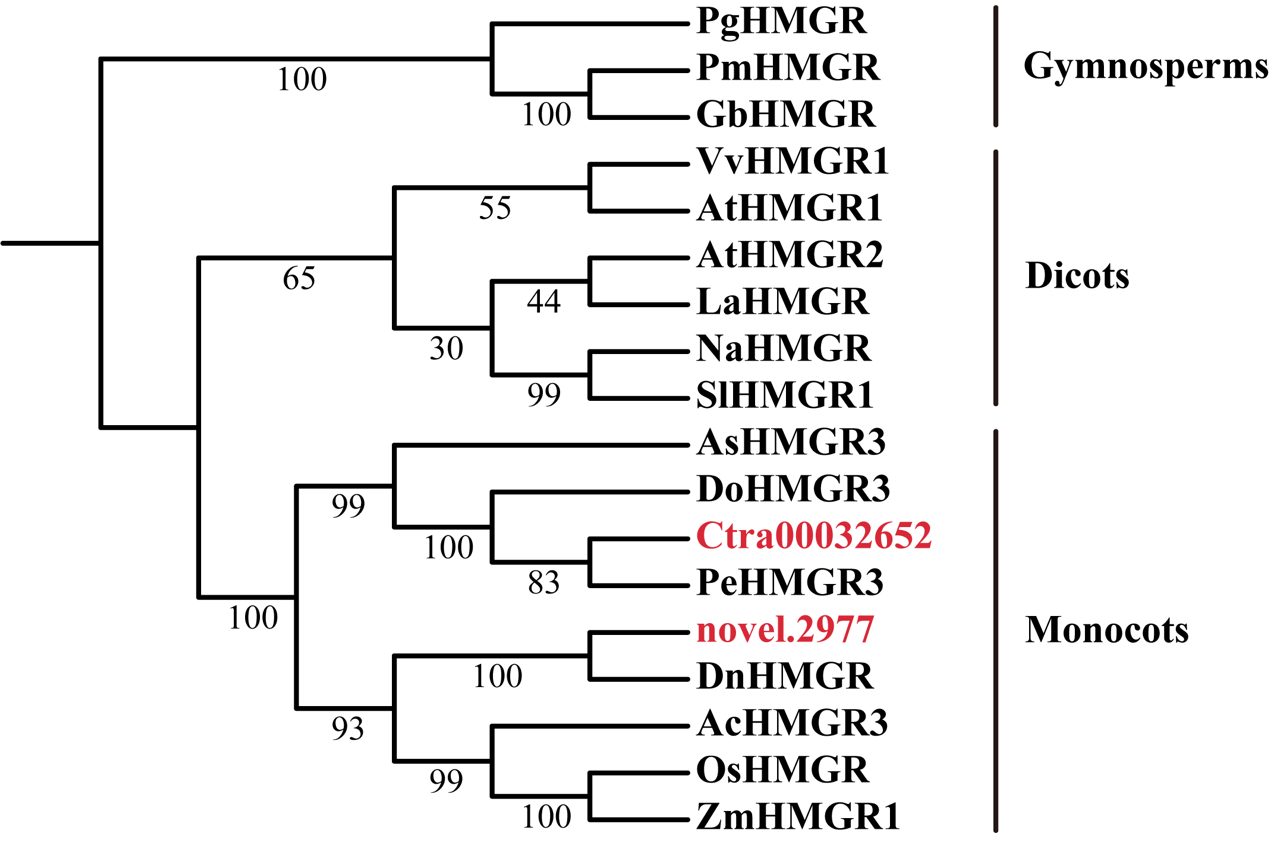


**Figure S11 Phylogenetic analysis of HMGR proteins from *C. tracyanum* and other plants.** The abbreviations, species, and NCBI accession numbers of HMGRs are as follows: PgHMGR, *Picea glauca*, JAI17633.1; PmHMGR, *Pinus massoniana*, QGZ18961.1; GbHMGR, *Ginkgo biloba*, AXV45367.1; VvHMGR1, *Vitis vinifera*, XP_002275827.1; AtHMGR1, *Arabidopsis thaliana*, NP_177775.2; AtHMGR2, *Arabidopsis thaliana*, AEC06618.1; LaHMGR, *Lavandula angustifolia*, AGQ04159.1; NaHMGR, *Nicotiana attenuata*, OIT06551.1; SlHMGR1, *Solanum lycopersicum*, NP_001296810.1; AsHMGR3, *Apostasia shenzhenica*, PKA52834.1; DoHMGR3, *Dendrobium catenatum*, XP_020705018.1; PeHMGR3, *Phalaenopsis equestris*, XP_020579399.1; DnHMGR, *Dendrobium nobile*, AOO95898.1; AcHMGR3, *Ananas comosus*, XP_020090296.1; OsHMGR, *Oryza sativa*, AAD08820.1; ZmHMGR1, *Zea mays*, ZmHMGR1.


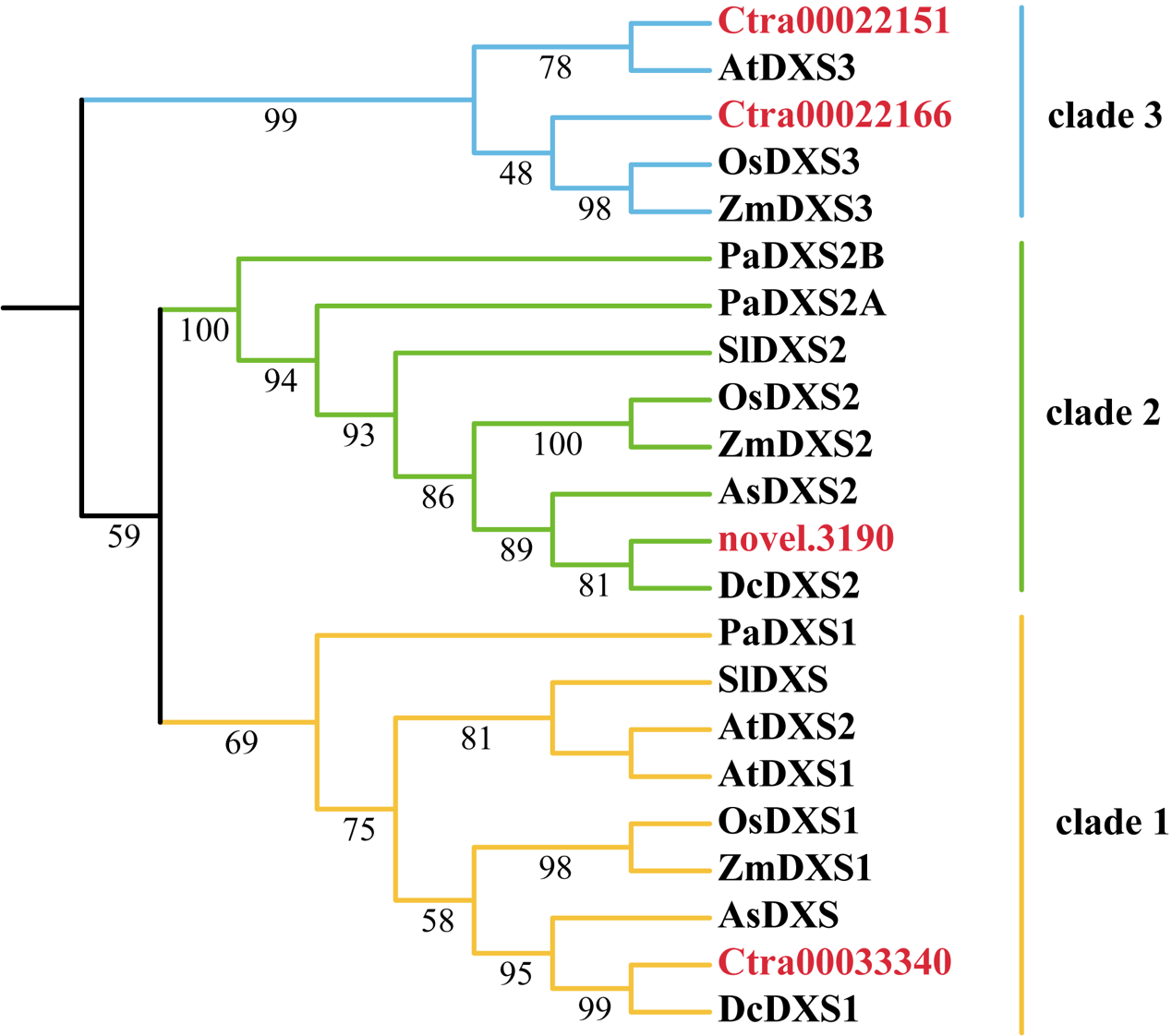


**Figure S12 Phylogenetic analysis of DXS proteins from *C. tracyanum* and other plants.** CtDXS proteins in this study are highlighted in red font. The abbreviations, species, and NCBI accession numbers of DXSs are as follows: AtDXS1, *Arabidopsis thaliana*, AAN86173.1; AtDXS2, *Arabidopsis thaliana*, NP_001327775.1; AtDXS3, *Arabidopsis thaliana*, NP_196699.1; ZmDXS1, *Zea mays*, NP_001157805.1; ZmDXS2, *Zea mays*, ABP88135.1; ZmDXS3, *Zea mays*, ADN22972.1; OsDXS1, *Oryza sativa*, NP_001055524.1; OsDXS2, *Oryza sativa*, NP_001059086.1; OsDXS3, *Oryza sativa*, BAA83576; PaDXS1, *Picea abies*, ABS50518; PaDXS2A, *Picea abies*, ABS50519.1; PaDXS2B, *Picea abies*, ABS50520.1; SlDXS, *Solanum lycopersicum*, NP_001234672.1; SlDXS2, *Solanum lycopersicum*, NP_001332799.1; AsDXS, *Apostasia shenzhenica*, PKA64821.1; AsDXS2, *Apostasia shenzhenica*, PKA60992.1; DcDXS1, *Dendrobium catenatum*, PKU85825.1; DcDXS2, *Dendrobium catenatum*, PKU86497.1.


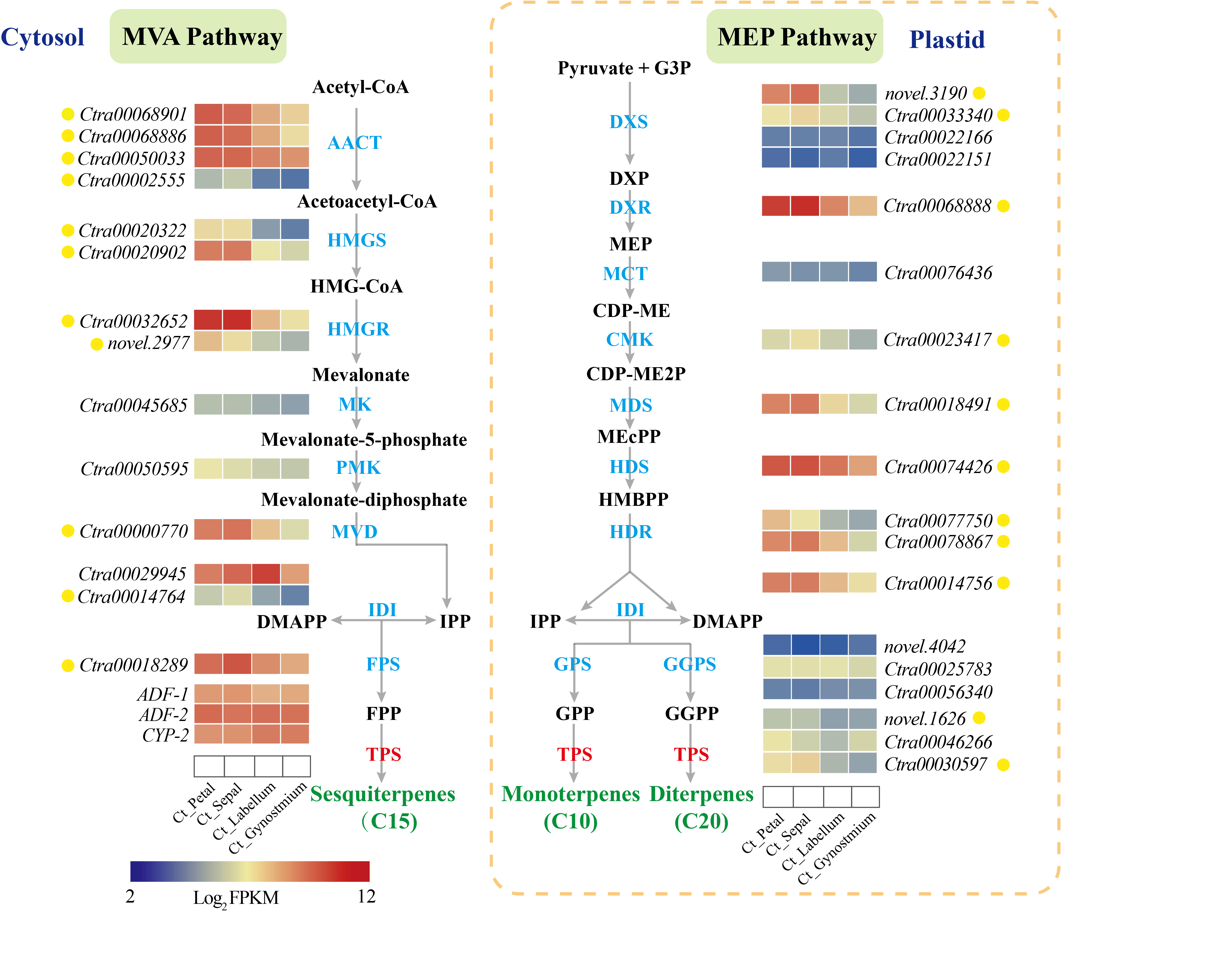


**Figure S13 Expression profiles of putative genes encoding enzymes for terpenoid biosynthesis in different parts of *C. tracyanum* flowers at full-blooming stage.** Abbreviations for enzymes in each catalytic step are highlighted in blue and red. The four boxes, from left to right, represent the gene expression levels in the petal, sepal, labellum, and gynostemium of *C. tracyanum*, respectively. Yellow dots indicate genes that are significantly highly expressed in petals and sepals compared to the labellum and gynostemium in *C. tracyanum* at the full-blooming stage (see Table S15).

**
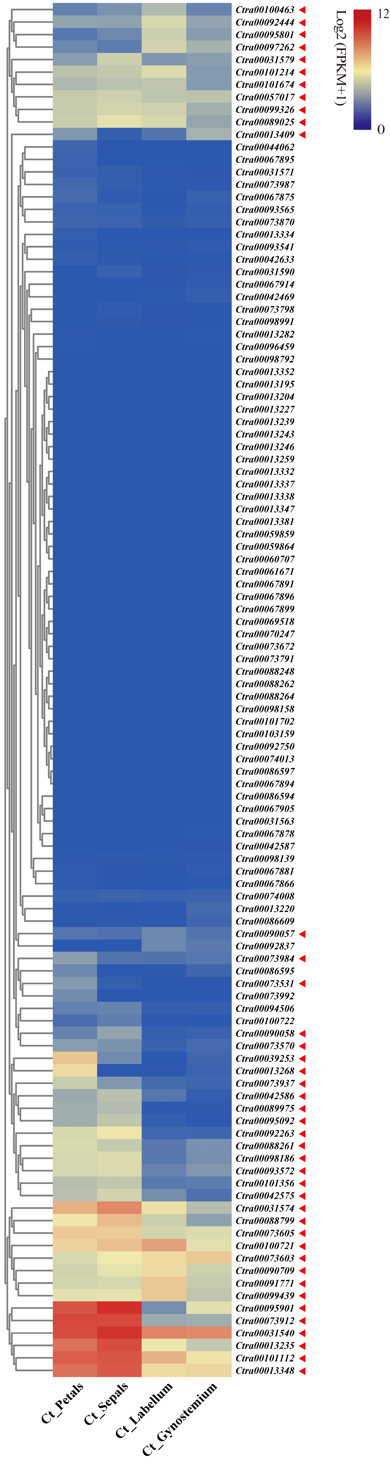
**

**Figure S14 Heatmap of the expression patterns of *TPS* genes from different parts of *C. tracyanum* flowers at the full-blooming stage.** The expression levels of each gene are represented as log_2_(FPKM+1) values. Genes marked with red triangles are those with an average FPKM > 1 in different floral parts at the full-blooming stage.

**Figure S15 Hierarchical cluster tree displaying 25 modules of co-expressed genes.**

The different colors under the dendrogram represent co-expressed modules identified through WGCNA.


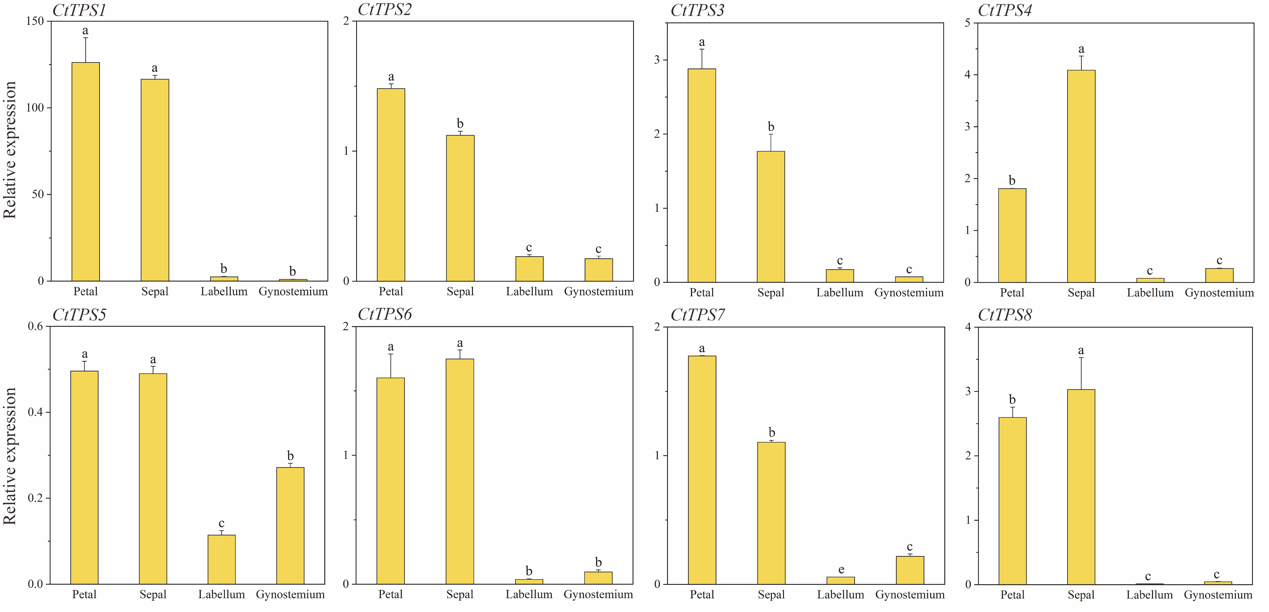


**Figure S16 Expression levels of eight *CtTPS* genes in different parts of *C. tracyanum* flowers (at the D+15 stage) by qRT-PCR analysis.** *CtGAPDH* is quantified as internal controls. Different letters indicate significant differences between floral parts determined by one-way ANOVA (*P* < 0.05). Error bars stand for the SD of three replicates.


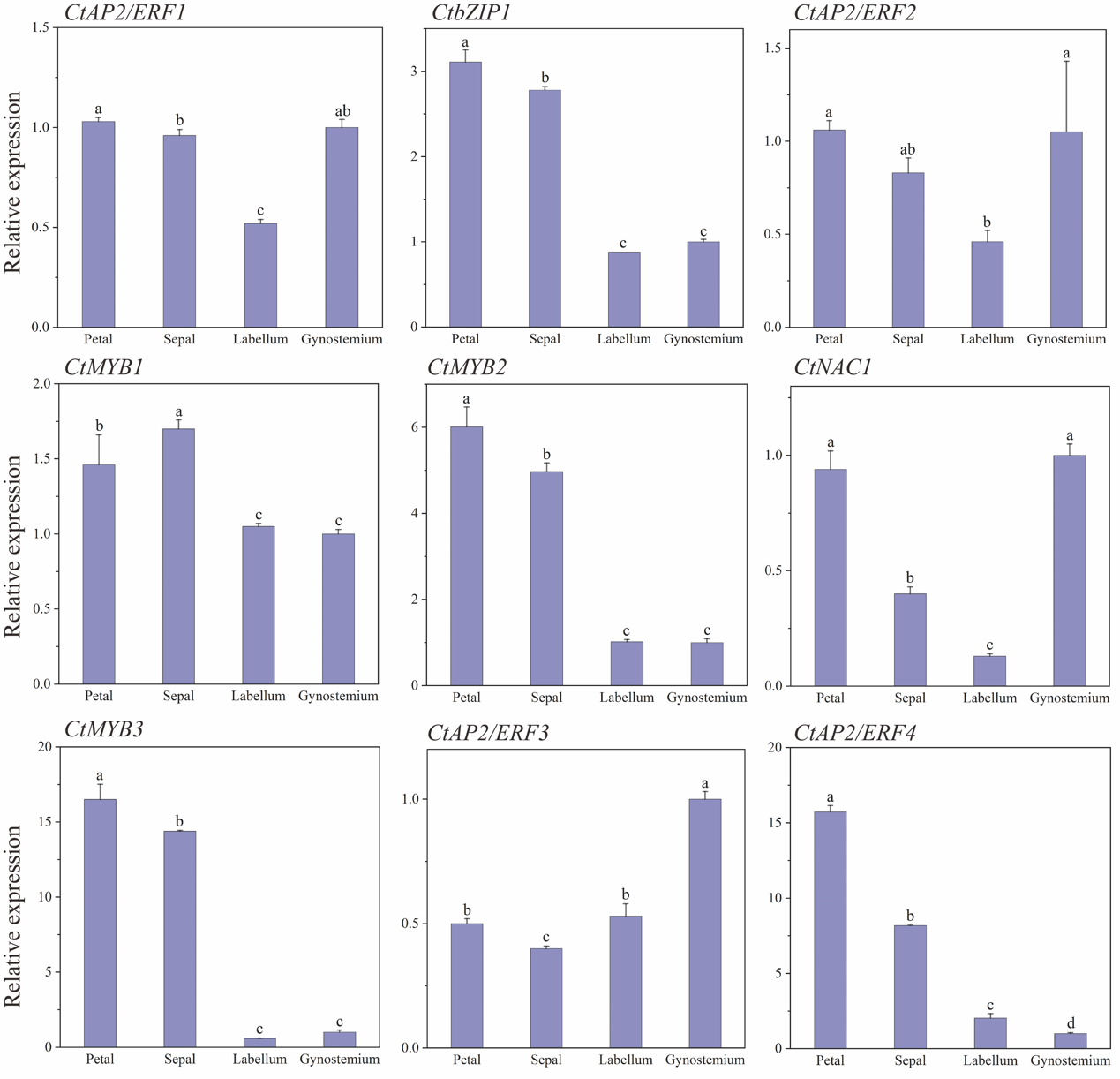


**Figure S17 Expression levels of candidate TFs in different parts of *C. tracyanum* flowers (at the D+15 stage) by qRT-PCR analysis.** *CtGAPDH* is quantified as internal controls. Different letters indicate significant differences between floral parts determined by one-way ANOVA (*P* < 0.05). Error bars stand for the SD of three replicates.


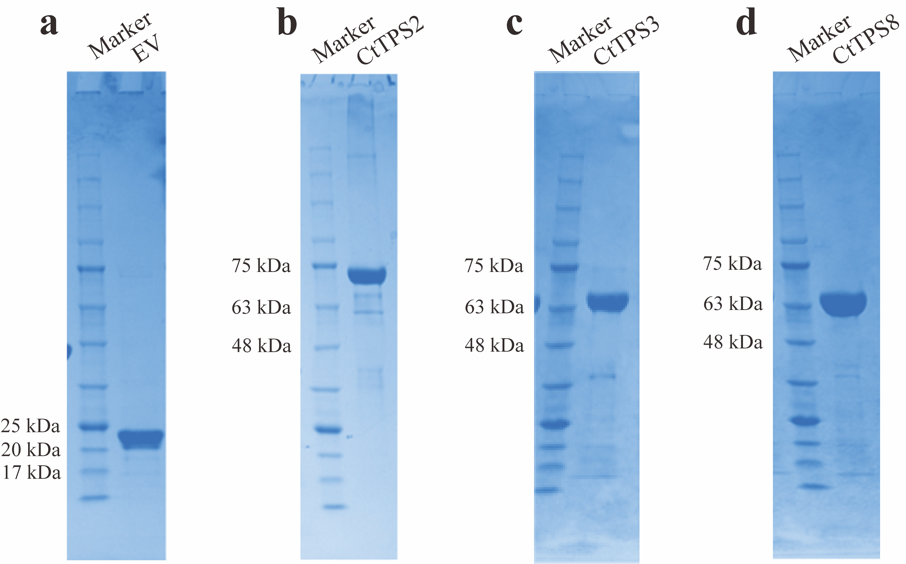


**Figure S18 SDS-PAGE analysis of recombinant terpene synthase proteins.** (a) pET-32a EV (calculated molecular mass 20.4 kDa); (b) CtTPS2 (calculated molecular mass 75.4 kDa); (c) CtTPS3 (calculated molecular mass 61.2 kDa); (d) CtTPS8 (calculated molecular mass 61.5 kDa).


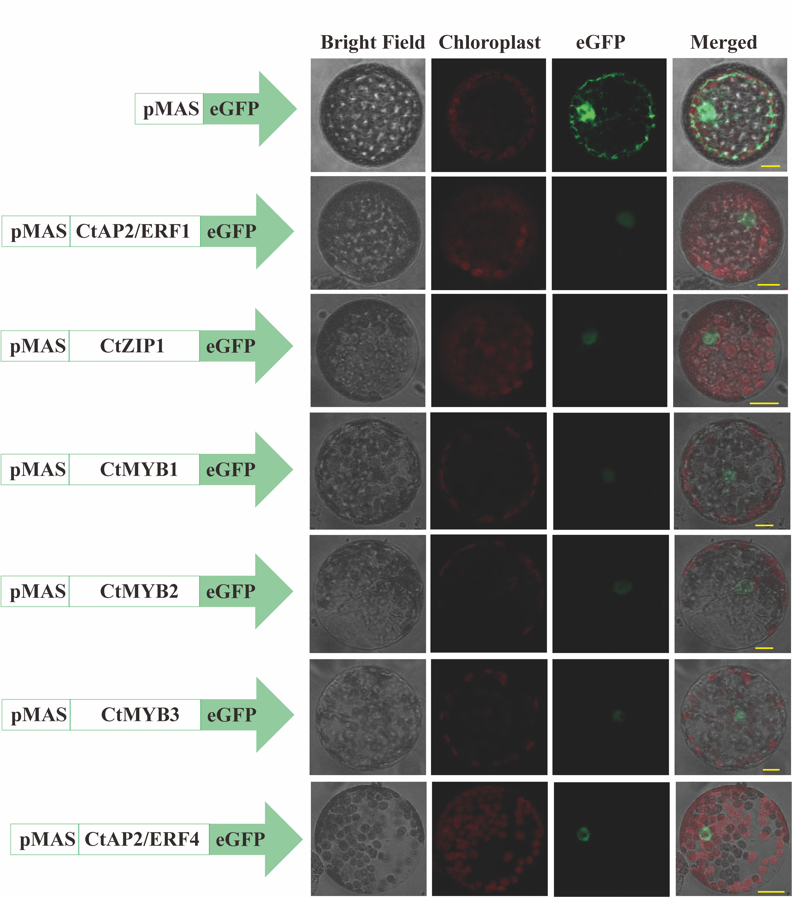


**Figure S19 Subcellular localization of free eGFP and six CtTF-eGFP fusions in tobacco leaf protoplasts.** CtTFs are targeted to the cell nucleus. The eGFP fluorescence detected in the green channel and chlorophyll autofluorescence detected in the red channel; Merged, brightfield image combined with green and red channels; Scale bar indicates 10 μm.


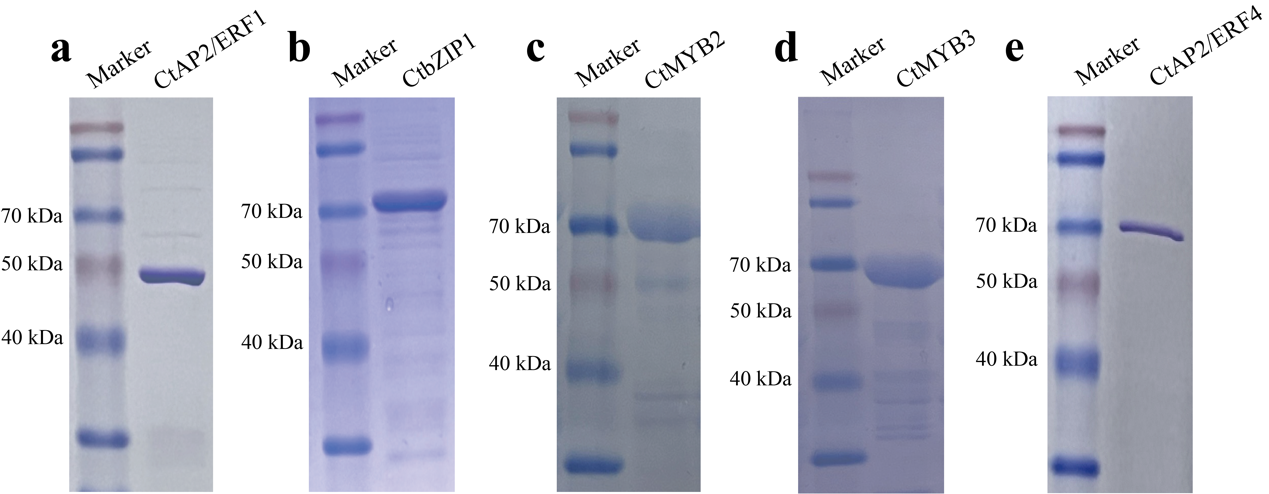


**Figure S20 SDS-PAGE analysis of recombinant transcription factor proteins.** (a) CtAP2/ERF1 (calculated molecular mass 53.1 kDa); (b) CtbZIP1 (calculated molecular mass 58.6 kDa); (c) CtMYB2 (calculated molecular mass 63.5 kDa); (d) CtMYB3 (calculated molecular mass 59.5 kDa); (e) Ct AP2/ERF4 (calculated molecular mass 57.4 kDa).


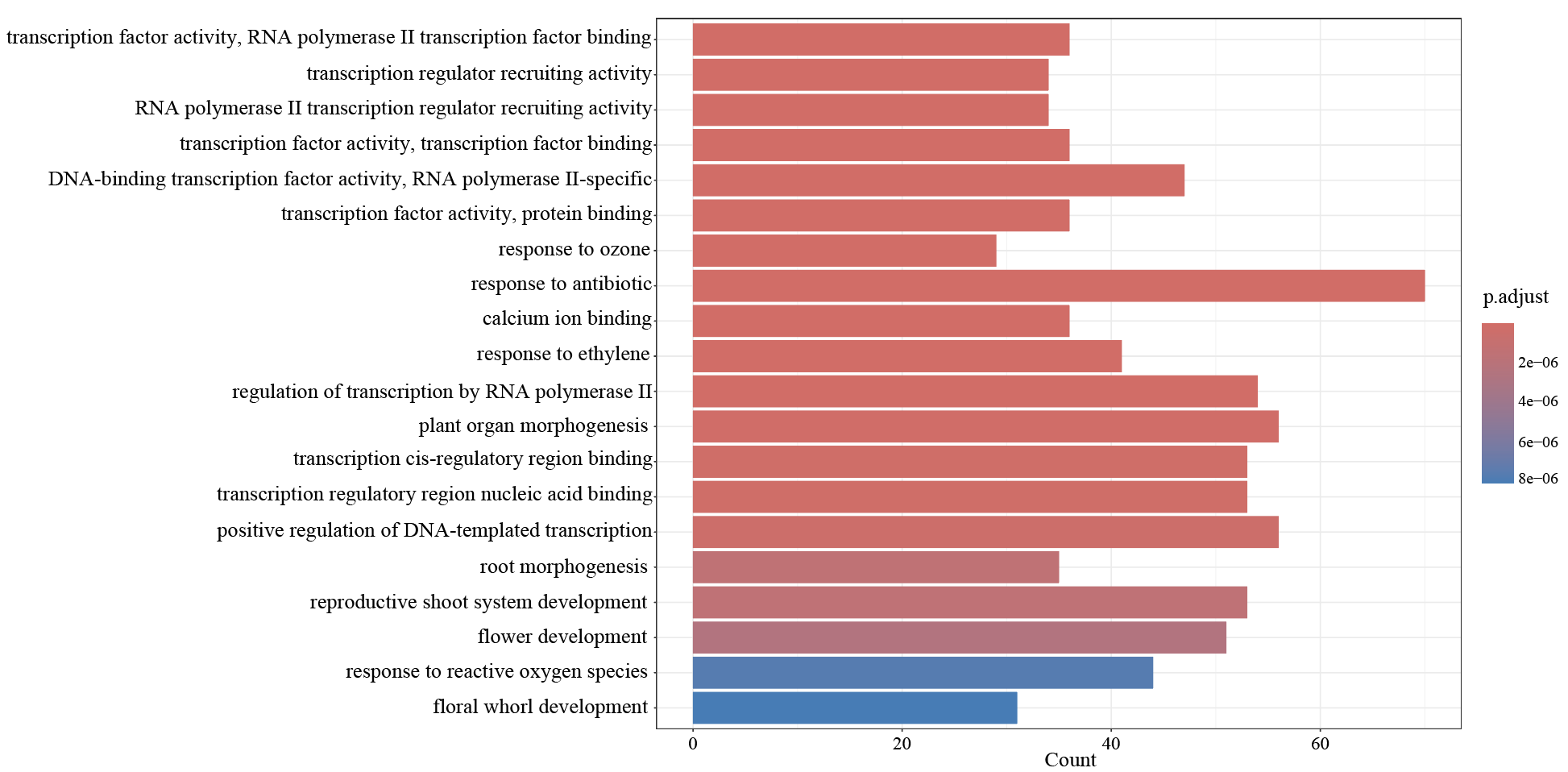


**Figure S21** **GO pathway enrichment distribution of the contracted genes in *C. tracyanum* genome.** GO enrichment analyses were based on the hypergeometric distribution, and *p*-values were adjusted for multiple hypothesis testing using the Benjamini-Hochberg (BH) method (padj). GO terms with padj < 0.05 were considered significant.
